# Drugs prescribed for Phelan-McDermid syndrome differentially impact sensory behaviors in *shank3 *zebrafish models.

**DOI:** 10.12688/f1000research.127830.2

**Published:** 2023-09-27

**Authors:** Robert A. Kozol, Julia E. Dallman

**Affiliations:** 1 Charles E. Schmidt College of Science, Florida Atlantic University, Jupiter, Fl., USA; 2 Department of Biology, University of Miami, Coral Gables, FL, 33146, USA

**Keywords:** Shank3, Phelan-McDermid Syndrome, autism spectrum disorders, zebrafish, Risperidone, Carbamazepine, Lithium, MPEP, anti-epileptic

## Abstract

**Background:** Altered sensory processing is a pervasive symptom in individuals with Autism Spectrum Disorders (ASD); people with Phelan McDermid syndrome (PMS), in particular, show reduced responses to sensory stimuli. PMS is caused by deletions of the terminal end of chromosome 22 or point mutations in *Shank3.* People with PMS can present with an array of symptoms including ASD, epilepsy, gastrointestinal distress, and reduced responses to sensory stimuli. People with PMS are often medicated to manage behaviors like aggression and/or self-harm and/or epilepsy, and it remains unclear how these medications might impact perception/sensory processing. Here we test this using zebrafish mutant *shank3ab* PMS models that likewise show reduced sensory responses in a visual motor response (VMR) assay, in which increased locomotion is triggered by light to dark transitions.

**Methods:** We screened three medications, risperidone, lithium chloride (LiCl), and carbamazepine (CBZ), prescribed to people with PMS and one drug, 2-methyl-6-(phenylethynyl) pyridine (MPEP) tested in rodent models of PMS, for their effects on a sensory-induced behavior in two zebrafish PMS models with frameshift mutations in either the N- or C- termini. To test how pharmacological treatments affect the VMR, we exposed larvae to selected drugs for 24 hours and then quantified their locomotion during four ten-minute cycles of lights on-to-off stimuli.

**Results:** We found that risperidone normalized the VMR in *shank3* models. LiCl and CBZ had no effect on the VMR in any of the three genotypes. MPEP reduced the VMR in wildtype (WT) to levels seen in *shank3* models but caused no changes in either *shank3* model. Finally, *shank3* mutants showed resistance to the seizure-inducing drug pentylenetetrazol (PTZ), at a dosage that results in hyperactive swimming in WT zebrafish.

**Conclusions:** Our work shows that the effects of drugs on sensory processing are varied in ways that can be highly genotype- and drug-dependent.

## Introduction

Altered sensory processing affects the majority (69-97%) of people with autism and is one of the core diagnostic symptoms in the Diagnostic and Statistical Manual V ( Leekam *et al*., 2007; Tomchek and Dunn, 2007; Lane *et al*., 2011; Green *et al*., 2016; Tavassoli *et al*., 2016; Siper *et al*., 2017). Such symptoms includes hypo- and hyper-reactivity to stimuli, and sensory fixation ( Robertson and Baron-Cohen, 2017). Consistent with this, genotype by symptom meta-analyses identified sensory hyporeactivity/increased-pain-tolerance in over 80% of individuals with Phelan-McDermid syndrome (PMS) ( Mieses *et al*., 2016; Tavassoli *et al*., 2016; De Rubeis, 2018). PMS is a syndromic form of ASD, that can be caused by a chromosome 22 terminal deletion that encompasses the *SHANK3* gene or a mutation in the *SHANK3* gene specifically ( Phelan and McDermid, 2012; De Rubeis, 2018). In addition to sensory hyporeactivity, *SHANK3* mutations are correlated with a range of symptoms, that include epilepsy, sleep disturbances, and gastrointestinal distress ( Soorya *et al*., 2013; De Rubeis, 2018; Frank, 2021; Smith-Hicks *et al*., 2021). This range of symptoms makes prescribing medications challenging ( Costales and Kolevzon, 2015; Harony-Nicolas *et al*., 2015), with many individuals experiencing a prescription carousel: when one drug fails to maintain control of a symptom and/or side-effects become intolerable. Therefore, to achieve more effective symptom management, it is critical to better understand how medications impact the range of symptoms found in individuals with PMS.

Zebrafish provide characteristics that are ideal for studying how small molecules impact sensory-motor behaviors. Zebrafish sensory-motor circuits are established and become active a few days after fertilization because precocial behavioral development is essential for the survival of freely swimming larvae ( Kimmel *et al*., 1974; Portugues and Engert, 2009; Fero *et al*., 2011; Kinkhabwala *et al*., 2011; Warp *et al*., 2012; Marques *et al*., 2018). Predator avoidance and prey capture require visual acuity, sensitive hearing, and multimodal sensory integration to activate the appropriate swimming circuits ( Fero *et al*., 2011; Koyama *et al*., 2011). Importantly, sensory-motor deficits provide a proxy for circuit pathology, that can be used to identify neuropathological critical periods ( Kozol, 2018; Sakai *et al*., 2018; Kozol *et al*., 2021). Finally, due to their small size and large clutch sizes (100-200 embryos), zebrafish can be screened in large numbers and also absorb most small molecules dissolved in the water that houses them. Therefore, zebrafish provide a vertebrate model that is poised to identify how small molecules influence sensorimotor behaviors in ASD models ( Sakai *et al*., 2018).

To investigate how drugs impact *SHANK3-*associated hyporeactivity, zebrafish *shank3a* and *shank3b* ( *shank3ab*) mutants were exposed to drugs and screened for sensorimotor behavior using a the well-established visual-motor-response (VMR) assay ( Burgess and Granato, 2007). During the VMR, sudden changes in illumination from light to dark evoke abrupt increases in swimming behavior as larvae search the well for a way to return to the light ( Horstick *et al.* 2017); we capture the abrupt response by quantifying swimming in the first 30 seconds right after the transition to dark, referred to hereafter as reactivity, but the larvae sustain their search for the full 5 minutes, referred to hereafter as activity. *shank3ab* mutants exhibit both hyporeactivity and sustained hypoactivity in response VMR repeated lights-on to lights-off transitions ( Kozol *et al*., 2021). To determine the effects of small molecules on this sensorimotor deficit, we exposed larval zebrafish to the commonly prescribed medications risperidone ( Nyberg *et al*., 1993; McDougle *et al*., 2005; Gencer *et al*., 2008; Lemmon *et al*., 2011), lithium chloride (LiCl) ( Malhi *et al*., 2013; Verhoeven *et al*., 2013; Serret *et al*., 2015; Egger *et al*., 2017; Malhi *et al*., 2020), and carbamazepine (CBZ) ( Mattson *et al*., 1992; Verhoeven *et al*., 2013; Jia *et al*., 2022). We also tested 2-methyl-6-(phenylethynyl) pyridine (MPEP), which normalized anxiety and striatal synaptic transmission in a *shank3* mouse model ( Wang *et al*., 2016). Lastly, we quantified swimming before and after exposure to pentylenetetrazole (PTZ), a drug used in animal models to better understand susceptibility to seizures, at doses that normally cause hyperactivity in wild type larvae ( Baraban *et al*., 2005; Hoffman *et al*., 2016; Liu and Baraban, 2019). Results of the above experiments are summarized in the column entitled ‘effect on VMR’ in Table 1.

**Table 1.** Drugs used in this study are listed to the left followed by indication and target(s)/mechanism of action. These are based on relevant references in the rightmost column. Drug effects on VMR in are based results from this study.

Drugs	Indication	Target(s)	Effect on VMR	Reference
Risperidone	Human Antipsychotic; Irritability in ASD	Various/unknown 5-HT _2C_; 5-HT _2A_; D _2_ a1/a2 adrenergic;H _1_ histamine receptor antagonists; Sodium channels	No change in WT; reduced VMR reactivity and rescued VMR sustained activity in *shank3ab-/-* models	( McDougle *et al*., 2005; Lemmon *et al*., 2011; Fallah *et al*., 2019; Panizzutti *et al*., 2021; Guber *et al*., 2022)
Carbamazepine CBZ	Human Anti-epileptic; Mood stabilizer	Various/unknown Sodium channels	VMR trended reduced in *shank3N* & WT No change in *shank3C*	( Mattson *et al*., 1992; Verhoeven *et al*., 2013; Jia *et al*., 2022)
LiCl	Human Mood stabilizer	Various: Dopamine; G-protein-coupled receptors; adenylate cyclase; phosphoinositide signals; MARKS, PKC, GSKb; GABA	No change in any genotype	( Malhi *et al*., 2013; Serret *et al*., 2015; Egger *et al*., 2017)
2-Methyl-6-(phenylethynyl) pyridine MPEP	Mouse models of Fragile X, Shank3	mGluR5	Reduced WT VMR to shank3 levels; no change in either *shank3ab-/-* model	( Tu *et al*., 1999; Tucker *et al*., 2006; Vucurovic *et al*., 2012; Wang *et al*., 2016)
Pentylenetetrazole PTZ	Zebrafish/mouse seizure-inducing drug	GABA _A_ receptor antagonist	Induced seizure-like activity in WT. Both *shank3ab-/-* models exhibit reduced response to PTZ	( Baraban *et al*., 2005; Dhamne *et al*., 2017; Liu and Baraban, 2019)

Below we describe the varied ways these drugs impacted the VMR sensorimotor behavior, from having no effect to suppressing or enhancing the VMR in a *shank3-*genotype-specific manner.

## Methods

### Ethics, fish maintenance and husbandry

**Figure 1.**
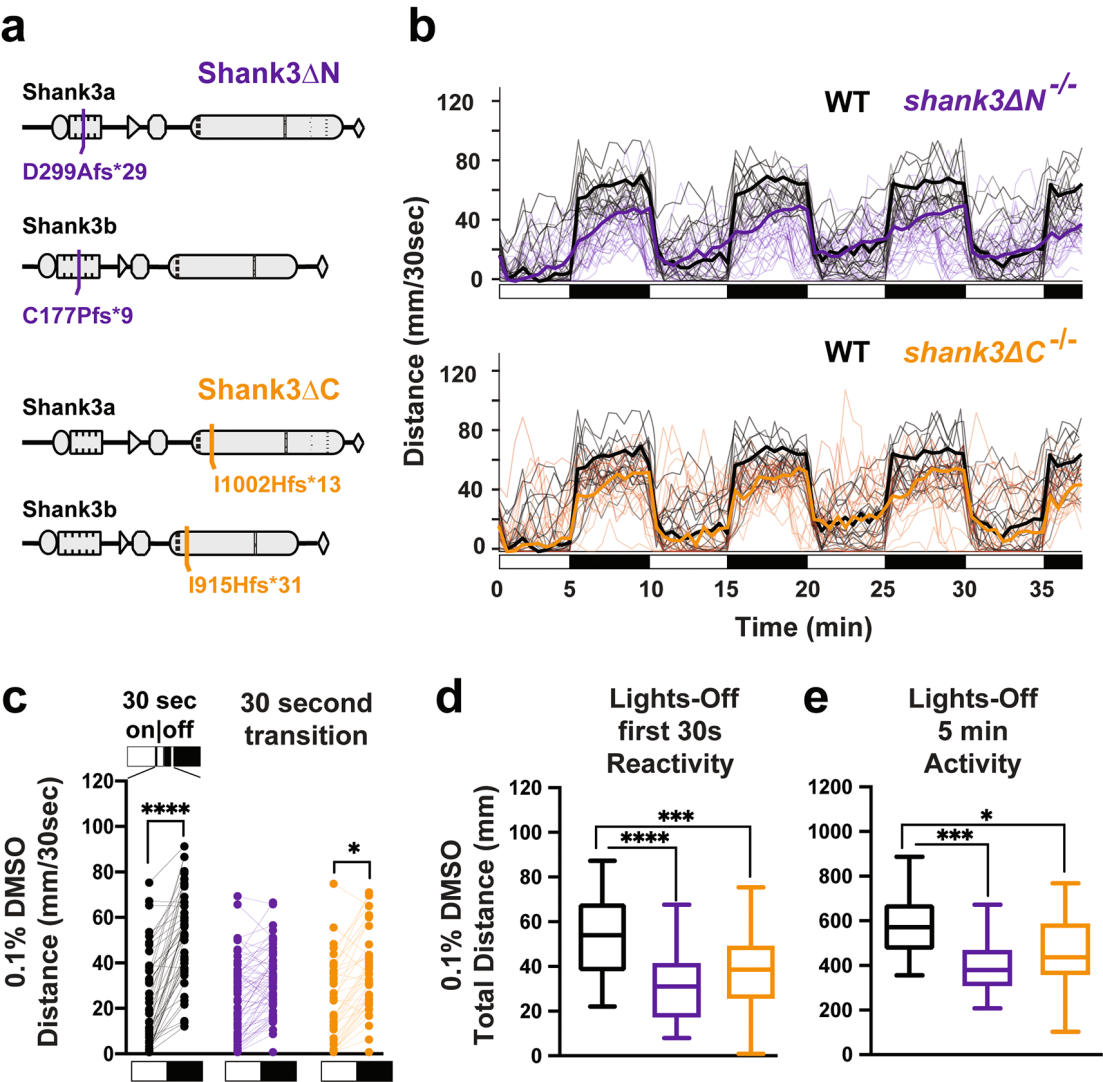
Stable *shank3ab* mutant lines exhibit hyporeactivity and hypoactivity following a light to dark transition. a) *shank3ab* N-terminal and C-terminal mutants were designed to target regions with known deleterious mutations in individuals with PMS. b) Trace line graphs showing four cycles of 5 minutes lights-on to lights-off. Checkered boxes on the x-axis represent lights on and off. c) Lights on to off paired comparison, highlighting no significant change in activity of *shank3ab* N terminal mutants during the first 30 sec lights-off. d) Box plots showing first 30 sec lights-off activity. e) Box plots showing activity across the full 5 minutes lights-off. Box plots represent 25 ^th^ and 75 ^th^ percentile, and median, with min to max whiskers. Sample sizes: WT = 50, *shank3* N = 65, *shank3* C = 44. p values; * = p < 0.05, ** = p < 0.01, *** = p < 0.001, **** = p < 0.0001.

Zebrafish were housed and maintained at 28 **°**C in system-water on a 14:10 hour circadian light:dark cycle in the zebrafish core facility at the University of Miami where they were fed twice a day using a combination of dry fish food and brine shrimp. The water in which the adult fish are housed are tested for pH and conductivity by probes that are always sampling, ‘system water’. System water is tap water that goes through a water softener, a charcoal filter, and reverse osmosis membranes to make the water less hard/alkaline, remove contaminants and ions respectively. This purified water is stored on a 100 gallon storage tank and used for 10% daily water exchanges that are controlled by a solenoid. pH 7.0-8.1 and conductivity 350-800 µS are kept within range by two dosers, one with sodium bicarbonate (pH) and the other with instant ocean (conductivity). We also track room humidity and temperature on a daily basis. These values are important to track because the temperature of the water is regulated by air temperature. Adult and larval zebrafish used in this study were handled in accordance with NIH guidelines and experiments were approved by the University of Miami Institutional Care and Use Committee protocol #’s 15-128 (approval date 9/22/2015) and 18-128 (approval date 9/27/2018). To limit harm to the animals and ensure experimental reproducibility, after natural spawnings, unfertilized eggs were removed and embryos were maintained in 10 cm dishes with ~50 larvae per dish until behavioral observations. Embryos were raised with the same 14:10 light cycle as their parents. Zebrafish lines used in this study were; ABTL wildtype (WT), *shank3abN-/-* ( Kozol *et al*., 2021) and *shank3abC-/-* ( James *et al*., 2019). Readers should note that each model includes a mutation in both the a and the b ohnolog of the *shank3* gene and therefor mutants are referred to as *shank3ab*; mutations in *shank3abN* are located near the N-terminus while those in *shank3abC* are located near the C-terminus of the predicted *Shank3* protein product ( Figure 1a).

This study is reported in line with the Animal Research: Reporting of in vivo Experiments (ARRIVE) guidelines ( Kozol & Dallman, 2023).

### Behavioral assays

*Sample*

All exact sample sizes can be found in the figure legends. Sample sizes were derived from a previous study based on the same VMR behavioral endpoint ( Kozol *et al*., 2021).

*High-throughput behavioral screens*

Experimental plans were developed and refined during weekly meetings but there was no protocol registered prior to initiation of experiments. The DanioVision system ^tm^ (Noldus, Wageningen, NTD) with the DanioVision observation chamber (DVOC-0040) was used to record videos of larval behaviors during experiments using the following settings: 25 fps, 1280 × 960 resolution using a Basler acA1300-60 gm camera fitted with a 12 mm Megapixel lens. White light for the visual motor response assay was set at 12% intensity on the high-power setting. Larvae were pipeted into an ANSI-SBS-compatible 96 well microtiter plate at a density of one larva per well, at a depth of 10 mm. Six-day-old larvae were acclimated to the observation chamber at 28 °C in the dark for at least 1 hr. Larval sex is unknown at this stage. Larvae were monitored during behavioral recordings, to ensure no signs of distress were exhibited during light cycles. DanioVision EthoVision XT software version 11.5 (Noldus) was used to set up data collection and for preliminary analyses. Visual motor response (VMR) experiments consisted of four cycles of alternating lights-on (five min.)/lights-off (five min.) for a total of 40 minutes. All behavioral experiments were conducted between 11 am and 3 pm, with 2-5 independent trials. Behavior was analyzed by binning the raw ethovision movement data into 30 second and 5 minute bins. We then defined behaviors in the first 30 seconds after dark transitions as reactivity and behaviors sustained across the full five minutes of darkness as activity. Therefore, a statistical increase or decrease in swimming during the first 30 seconds was defined as hyperreactive or hyporeactive respectively; a statistical increase or decrease in swimming during the full five minutes was defined as hyperactive or hypoactive respectively. Larvae were randomly assigned across each 96-well plate, blinded to experimenters, then were genotyped following behavioral experiments using restriction digest assays previously described ( James *et al*., 2019; Kozol *et al*., 2021), allowing larvae to be binned by genotype for subsequent analyses. Following experiments, larvae were humanely euthanized using MS222 (200 mg/L dissolved in system water).

*Drug screening*

Zebrafish were exposed to drugs dissolved in 0.1% DMSO system water (water from the system that houses the adult fish) 24 hours prior to running VMR assays. A range of risperidone, MPEP, CBZ and LiCl concentrations were derived from previously published papers ( Tucker *et al*., 2006; Bruni *et al*., 2016; Hoffman *et al*., 2016), then dose-response curves were generated to determine an effective dose in relation to the VMR response of WT zebrafish. Concentrations used for comparing WT and *shank3* larvae were 10 μM Risperidone ( Bruni *et al*., 2016; Hoffman *et al*., 2016), 5 mM LiCl and 200 μM CBZ, and 5 μM MPEP ( Tucker *et al*., 2006). Genotype controls were exposed to DMSO (0.1%) in system water.

For PTZ trials, larvae were initially acclimated in 1 mL of system water at 28 °C in the Daniovision behavioral box for 30 minutes. Larvae were then recorded for 10 minutes to establish baseline behavior. Following a baseline recording, larvae were either exposed to 3 mM PTZ in 0.1% DMSO system water or 0.1% DMSO system water for ten minutes, before capturing ten minutes of behavior following drug exposure. Baseline and PTZ/DMSO data was then binned as total distance moved for 10 minutes pre and post PTZ exposure. Both heterozygote and homozygote larvae were tested for seizure susceptibility, however to remain consistent with the other genotypes analyzed in the study, we chose to focus on the homozygote data.

### Statistics

Data were analyzed using PRISM 9 (graphpad, inc.); these same analyses could be conducted using R. Videos were manually screened before running data analyses, to determine that tracking software accurately captured individuals’ movements; if discrepancies between tracks and videos were noted, videos were retracked. No individuals or data points were excluded from behavioral analyses. Significance was assessed using the non-parametric Wilcoxon rank score test (Mann-Whitney rank scores). When there were more than two groups, a Kruskal-Wallis rank score test was first calculated and, if p<0.05, was followed by a Dunn’s multiple comparisons test to compare all treatments and genotypes. See Tables 2- 41.

## Results

### Zebrafish *shank3ab* mutants are hypoactive and hyporeactive in response to lights-off transitions

We previously showed that both *shank3abN* and *shank3abC* mutants exhibit sensory hyporeactivity (activity during first 30 seconds in dark) and hypoactivity (activity over full 5 minutes in dark) in a light to dark transition paradigm, the VMR assay ( Kozol *et al.*, 2021). Here we repeat this assay, but this time in the presence of the drug carrier 0.1% DMSO. In comparison to WT ( Figure 1a & b, Tables 2 & 3), both *shank3abN-/-* and *shank3abC-/-* models exhibited hyporeactivity and hypoactivity ( Figure 1c-e, Tables 4- 7). These results provide a reliable sensorimotor phenotype that can be quantified following exposure to selected drugs ( Kozol & Dallman, 2023).

### Dose-response curves to identify effective doses for each small molecule

**Figure 2.**
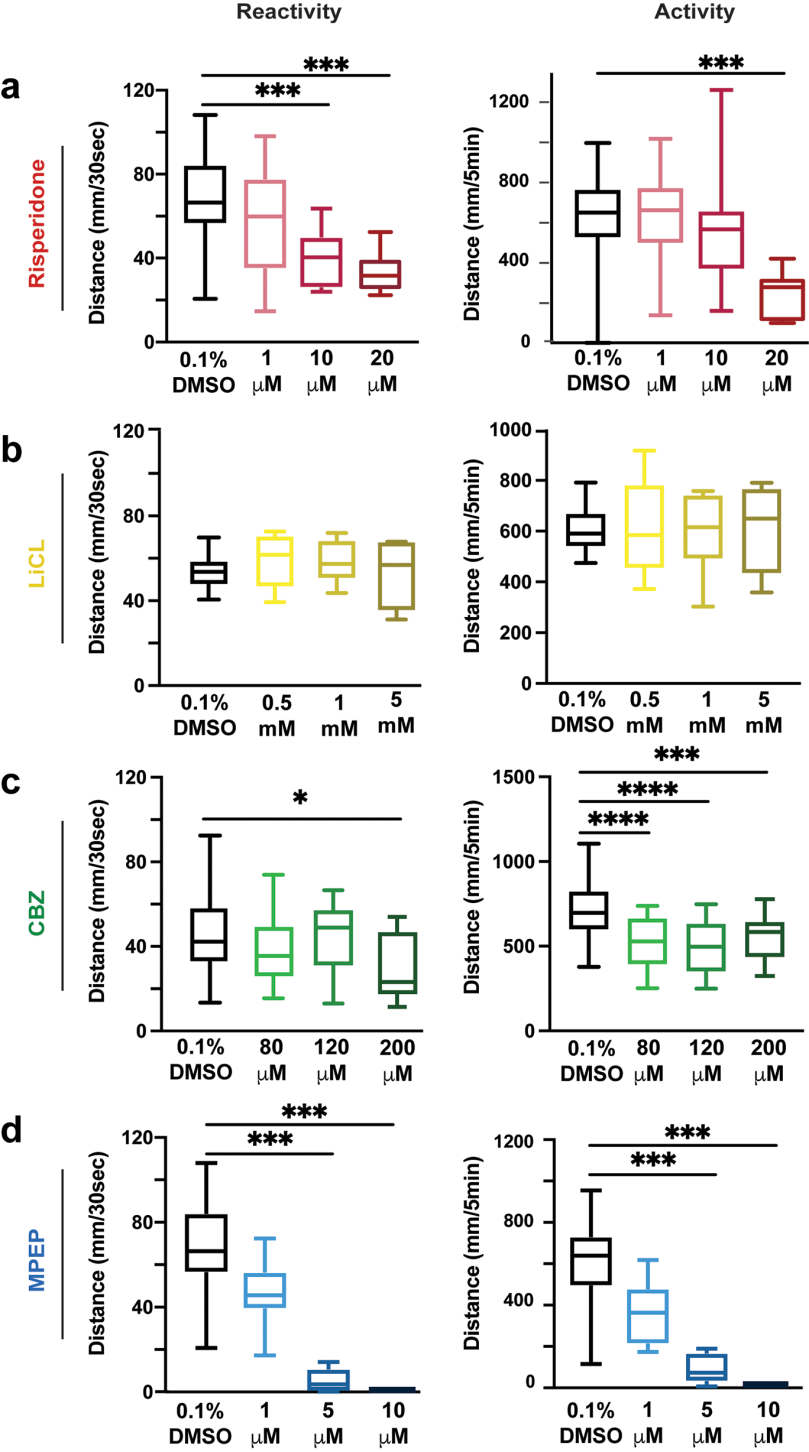
Dose response curves for drugs used in visual motor response assays. a) Risperidone exposure of WT larvae in 1, 10 and 20 μM doses. b) LiCl salt exposure of 0.5, 1 and 5 mM doses. c) CBZ exposure of WT larvae in 80, 120 and 200 μM doses. d) MPEP exposure of WT larvae in 1, 5 and 10 μM doses. Box plots represent 25 ^th^ and 75 ^th^ percentile, and median, with min to max whiskers. Sample sizes: WT = 23, WT + risperidone = 24, *shank3* N = 33, *shank3* N + risperidone = 31, *shank3* C = 19, *shank3* C + risperidone = 23. p values; * = p < 0.05, ** = p < 0.01, *** = p < 0.001, **** = p < 0.0001.

Dose-response curves for small molecules were performed to investigate how these drugs impact the VMR in WT larvae. Risperidone did not affect the VMR at 1 μM, while at 10 and 20 μM doses, the VMR was decreased ( Figure 2a, Tables 8- 11). LiCl did not impact the VMR in WT larvae, despite exceeding previously published concentrations ( Figure 2b, Tables 12- 13). In contrast, CBZ had varying effects on both reactivity and activity: 80 μM and 120 μM CBZ concentrations showed no effect a, while 200 μM caused larvae to be hypo-reactive ( Figure 2c, Tables 14- 17). Similarly, 1 μM of MPEP did not affect the VMR, while 5 and 10 μM the VMR was decreased ( Figure 2d, Tables 18- 21). These results provide the lowest effective concentrations for each drug, risperidone (10 μM), CBZ (200 μM) and MPEP (5 μM), that caused a significant decrease in WT activity and reactivity; for LiCl we proceeded with the high dose of 5 mM. We next used these small molecule concentrations to compare how each would impact sensorimotor behavior in *shank3ab-/-* mutants.

### Risperidone normalizes lights-off hypoactivity in *shank3ab* mutants

**Figure 3.**
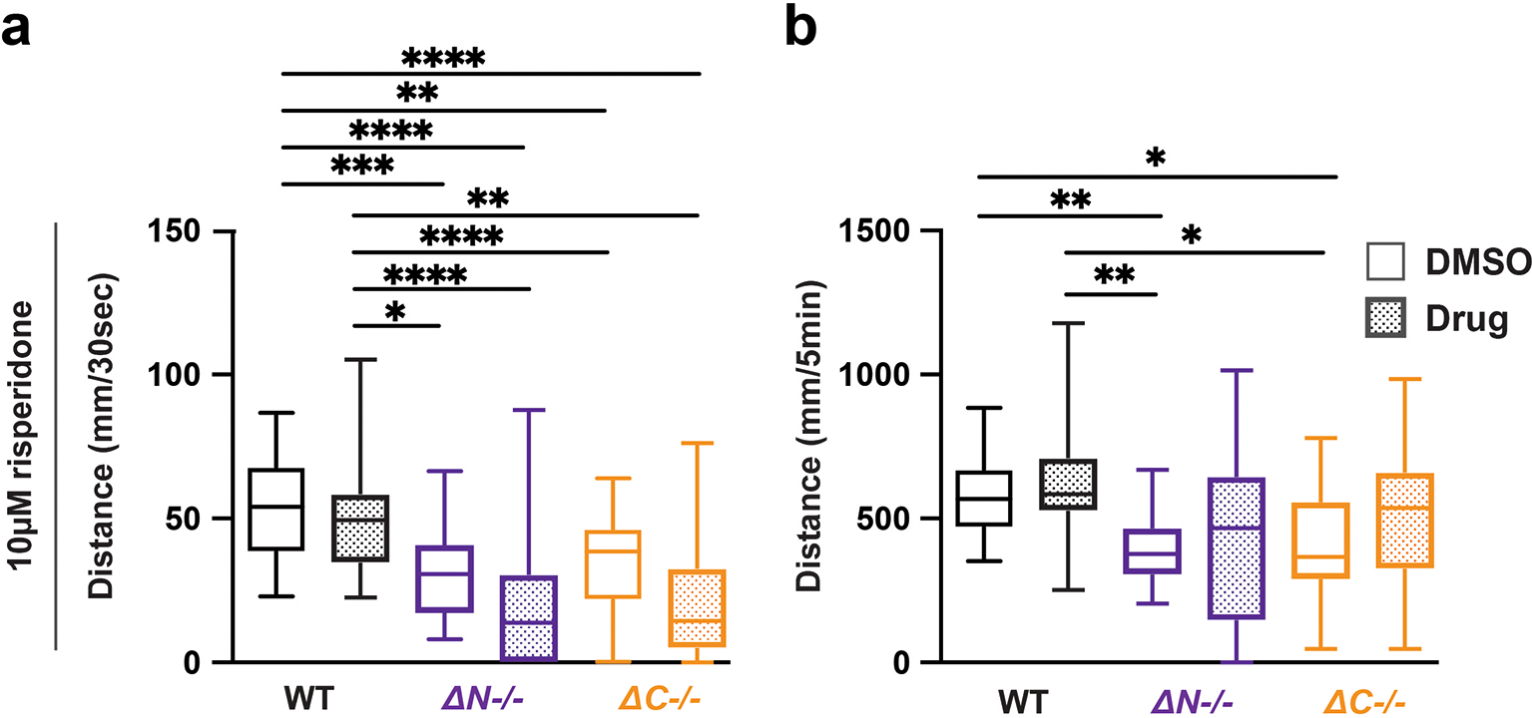
Risperidone exposure normalizes hypoactivity in *shank3* mutants following lights-off. a) Activity during the first 30 seconds of lights-off of larvae exposed to 10 μM risperidone. b) Activity during the full 5 minutes lights-off of larvae exposed to 10 μM risperidone. Box plots represents 25 ^th^ and 75 ^th^ percentile, and median, with min to max whiskers. Sample sizes: WT = 31, WT + risperidone = 29, *shank3* N = 24, *shank3* N + risperidone = 23, *shank3* C = 25, *shank3* C + risperidone = 23. p values; * = p < 0.05, ** = p < 0.01, *** = p < 0.001, **** = p < 0.0001.

Risperidone is commonly prescribed in ASD for aggressive, self-injurious and hyperactive behavior ( Lemmon *et al*., 2011). In *shank3ab-/-* mutants, 10 μM risperidone exacerbated hyporeactivity, but normalized hypoactivity, with *shank3ab* mutants achieving wild-type levels of swimming over the full duration of lights-off conditions ( Figure 3, Tables 22- 25). These results show that risperidone both reduced *shank3* stimulus reactivity, and normalized overall stimulus-driven behaviors in *shank3ab* mutants.

### LiCl does not impact light evoked sensorimotor behavior in *shank3* mutants or wildtype

**Figure 4.**
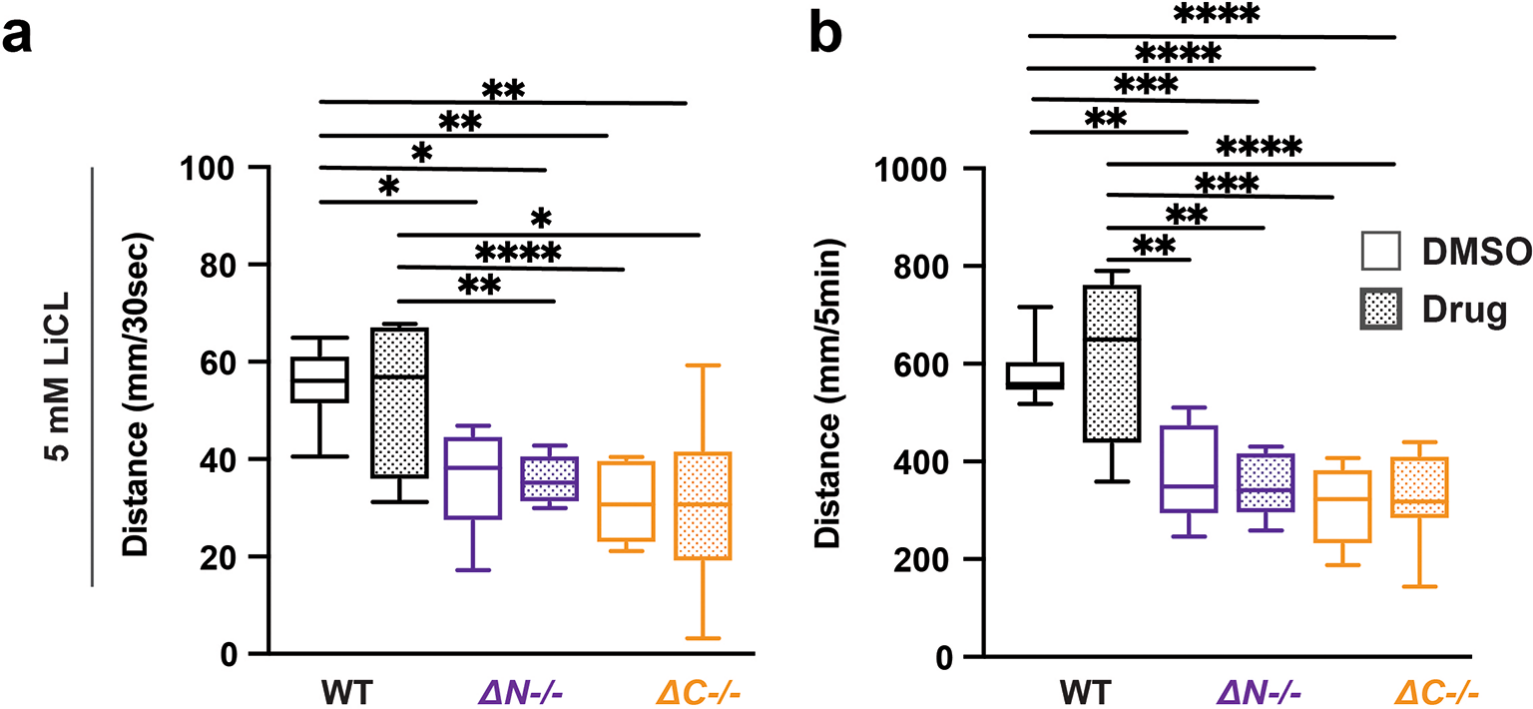
LiCl does not impact lights-off reactivity or activity in wildtype and *shank3ab* mutants. a) Activity during the first 30 seconds of lights-off of larvae exposed to 5 mM LiCl. b) Activity during the full 5 minutes lights-off of larvae exposed to 5 mM LiCl. Box plots represent 25 ^th^ and 75 ^th^ percentile, and median, with min to max whiskers. Sample sizes: WT = 20, WT + risperidone = 16, *shank3* N = 18, *shank3* N + risperidone = 18, *shank3* C = 16, *shank3* C + risperidone = 16. p values; * = p < 0.05, ** = p < 0.01, *** = p < 0.001, **** = p < 0.0001.

Lithium chloride (LiCl) has been prescribed for several neuropsychological disorders, including bipolar disorder, depression, and ASD ( Malhi *et al*., 2020). LiCl has been prescribed to individuals with PMS that exhibit bipolar depression, psychosis, and catatonic behavior ( Verhoeven *et al*., 2013; Egger *et al*., 2017). Exposure to 5 mM LiCl caused no change in *shank3ab-/-* VMR ( Figure 4, Tables 26- 29). Therefore, LiCl does not impact visual processing in either WT zebrafish or *shank3* mutant larvae.

### Carbamazepine does not impact light evoked sensorimotor behavior in *shank3* mutants or wildtype

**Figure 5.**
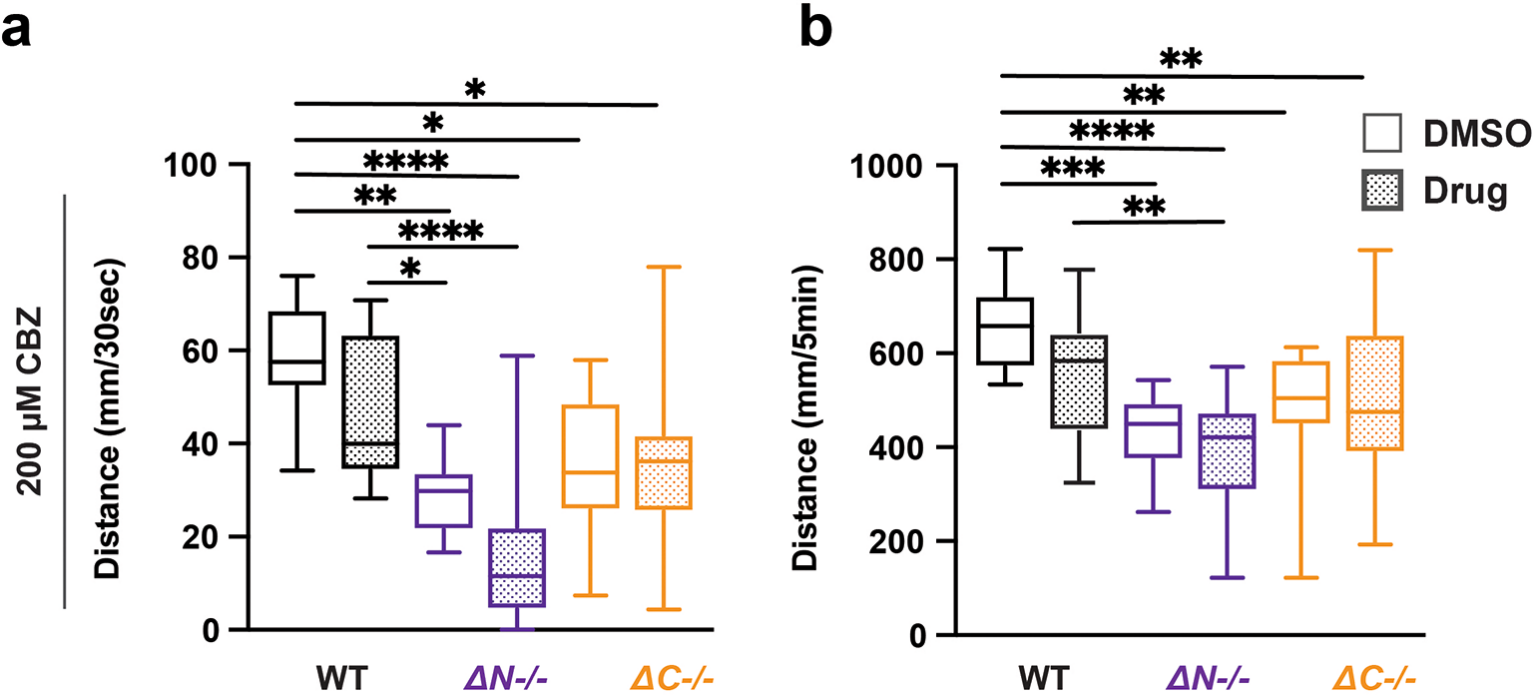
CBZ does not impact lights-off reactivity or activity in wildtype and *shank3ab* mutants. a) Activity during the first 30 seconds of lights-off of larvae exposed to 200 μM CBZ. b) Activity during the full 5 minutes lights-off of larvae exposed to 200 μM CBZ. Box plots represent 25 ^th^ and 75 ^th^ percentile, and median, with min to max whiskers. Sample Sizes, WT = 35, WT + CBZ = 37, *shank3ab* N = 24, *shank3ab* N + CBZ = 19, *shank3ab* C = 27, and *shank3ab* C + CBZ = 34. p values; * = p < 0.05, ** = p < 0.01, *** = p < 0.001, **** = p < 0.0001.

Carbamazepine (CBZ) is commonly prescribed to control seizures in individuals with epilepsy ( Mattson *et al*., 1992). For individuals with PMS, CBZ has been prescribed following symptom resistance to common mood stabilizers, such as lithium and valproic acid ( Verhoeven *et al*., 2013). WT and *shank3abN-/-* mutants VMR reactivity trended reduced with CBZ exposure but did not reach p < 0.05 ( Figure 5, Tables 30- 33). By contrast, *shank3ab* C-terminal VMR reactivity was unaffected by CBZ exposure. These results suggest that CBZ could have differential impacts on sensorimotor circuits depending on the location of the mutation in the *shank3* gene.

### Wildtype zebrafish recapitulated *shank3* mutant hypoactivity and hyporeactivity when exposed to the mGlur5 antagonist MPEP

**Figure 6.**
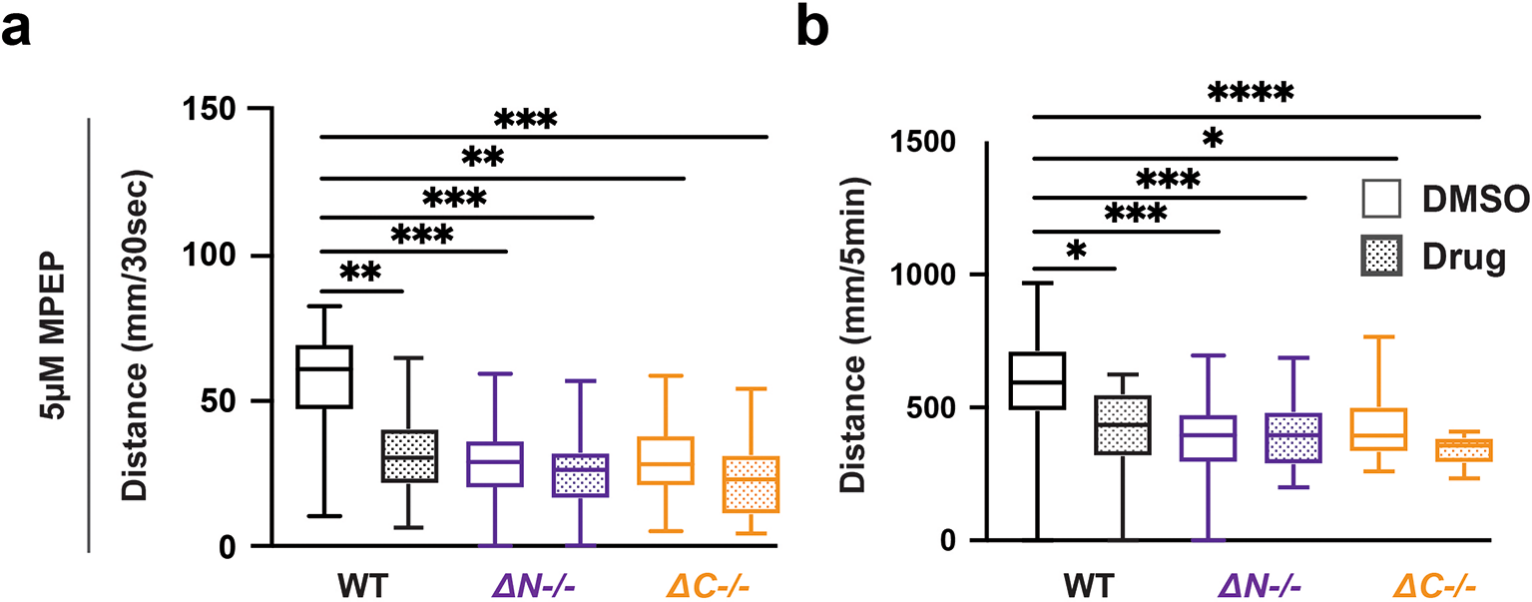
MPEP exposed wildtype larvae exhibit hyporeactivity and hypoactivity during lights-off conditions. a) Activity during the first 30 seconds of lights-off of larvae exposed to 5 μM MPEP. b) Activity during the full 5 minutes lights-off of larvae exposed to 5 μM MPEP. Box plots represent 25 ^th^ and 75 ^th^ percentile, and median, with min to max whiskers. Sample Sizes, WT = 23, WT + MPEP = 24, *shank3ab* N = 33, *shank3ab* N + MPEP = 31, *shank3ab* C = 19, and *shank3ab* C + MPEP= 24. p values; * = p < 0.05, ** = p < 0.01, *** = p < 0.001, **** = p < 0.0001.

While the molecules described above have been prescribed for ASD and epilepsy, we were also interested in investigating compounds used to rescue behavioral deficits in *Shank3* mouse models ( Wang *et al*., 2016). We found that MPEP did not affect the VMR in *shank3ab* mutants however, MPEP was sufficient to cause hyporeactivity and hypoactivity in WT larvae ( Figure 6, Tables 34- 37). Therefore effects of MPEP on sensory-induced behaviors were genotype-dependent.

### *shank3abN* and C homozygous mutants do not exhibit hyperactive swimming in response to the GABA _A_ receptor antagonist pentylenetetrazole

**Figure 7.**
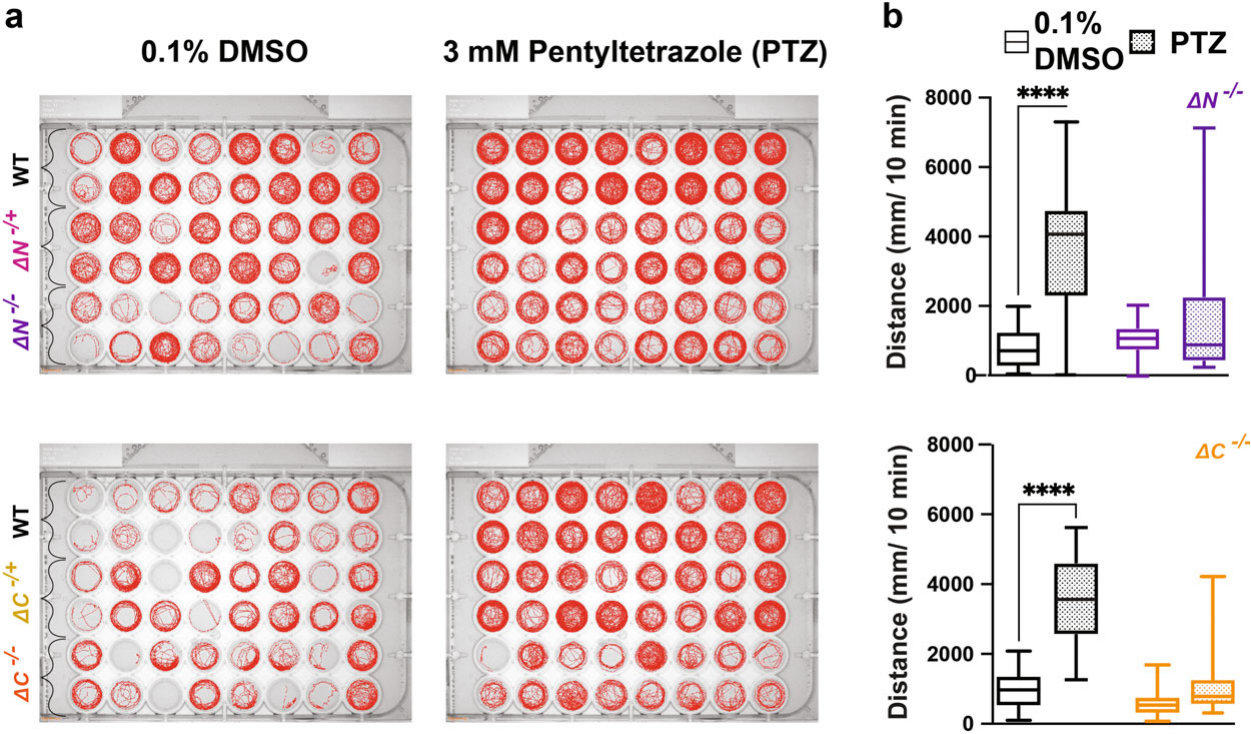
PTZ exposure does not induce seizure-like behavior in shank3 mutants. a) Behavioral traces of larvae exposed to 3 mM PTZ. b) Activity of WT, *shank3ab* N and *shank3ab* C larvae for 10 minutes following exposure to 3 mM of PTZ. Box plots: box represents 25 ^th^ and 75 ^th^ percentile, and median, with min to max whiskers. Sample sizes for *shank3* N trials, WT = 30, *shank3* N = 30; for *shank3* C trials, WT = 31, *shank3* C = 28. p values; * = p < 0.05, ** = p < 0.01, *** = p < 0.001, **** = p < 0.0001.

A standard approach used in animal models to test for susceptibility to seizures related to reduced GABAergic inhibition is to test responses to the GABA _A_ receptor antagonist pentylenetetrazole (PTZ) ( Baraban *et al*., 2005; Hoffman *et al*., 2016; Liu and Baraban, 2019). In response to 3 mM PTZ, both N and C *shank3ab-/-* larvae fail to exhibit WT level of hyperactivity suggesting altered GABAergic signaling in the *shank3ab* mutant models ( Figure 7, Tables 38- 41).

## Discussion

Here we show both drug- and genotype- specific effects on sensory-evoked VMR behavior in *shank3ab* zebrafish models of Phelan-McDermid Syndrome. The array of symptoms experienced by people with Phelan-McDermid Syndrome is likely a consequence of the diverse developmental and physiological roles played by *SHANK3* ( Sheng and Kim, 2000; Grabrucker, 2014; Harony-Nicolas *et al*., 2015; Kozol *et al*., 2015, 2021; Harris *et al*., 2016; Engineer *et al*., 2018; James *et al*., 2019; Breen *et al*., 2020; Lutz *et al*., 2020). Here we tested how drugs targeting aggressive behavior, catatonia, and/or epilepsy affect sensorimotor VMR behaviors in zebrafish *shank3* models of PMS. We found that drugs were neutral, enhanced or suppressed sensory-induced behavior in a genotype- and drug-dependent manner.

Zebrafish, in particular, provide a cost-effective and high-throughput way to test how medications impact behaviors ( Rihel *et al*., 2010; Kokel and Peterson, 2011; Rihel and Schier, 2013; Jordi *et al*., 2015; Bruni *et al*., 2016; Hoffman *et al*., 2016). We previously validated *shank3ab* N and C zebrafish models and showed a *shank3ab* mutant dose-dependent reduction in the VMR ( James *et al*., 2019; Kozol *et al*., 2021). Because the VMR phenotype is strongest in *shank3ab* homozygous larvae, we focused on this genotype for our small drug screen.

Widely-prescribed, mood-stabilizing medications risperidone and LiCl had distinct effects on the VMR. Risperidone exacerbated *shank3* VMR hyporeactivity and rescued overall activity to WT levels; by contrast, LiCl had no effect on the VMR in any of the three genotypes tested. In addition to the beneficial effects of risperidone however, this medication is associated with weight-gain in humans and reduced gastrointestinal motility in zebrafish ( de Alvarenga *et al*., 2017; Guber *et al*., 2022). Consistent with this, risperidone D _2_ and 5-HT _2_ receptor targets ( Nyberg *et al*., 1993) are expressed and regulate function in both brain and gut ( Taniyama *et al*., 2000; Eliassi *et al*., 2008; Feng *et al*., 2020). Therefore, risperidone creates known symptom trade-offs in addition to improving mood in people and visual processing in zebrafish.

Treatment-resistant epilepsy in Phelan-McDermid Syndrome is one of the most difficult symptoms to manage and also one for which there are many drug options ( Chakraborty *et al*., 2022). CBZ, a sodium channel blocker, has been used in patients with PMS who were resistant to mood stabilizers ( Mattson *et al*., 1992; Verhoeven *et al*., 2013, 2020). CBZ reduced reactivity to dark transitions in WT and *shank3abN-/-* larvae (though VMRs in neither genotype reached p<0.05) but had no effect on median VMR values in *shank3abC-/-* larvae, indicating possible *shank3* allele-specific differences in the way CBZ impacts the VMR. Consistent with *shank3* allele-specific differences, whole brain activity mapping in these same models showed a greater activity in mid and hindbrain circuits in response to dark transition in *shank3abN* than *shank3abC* alleles ( Kozol *et al*., 2021). Another drug that addresses seizure susceptibility is PTZ, a GABA _A_ receptor antagonist that is used to test seizure susceptibility in zebrafish and murine models. Our findings that *shank3ab* models are resistant to doses that make WT larvae hyperactive suggest that these models might have fewer GABA _A_ receptors targets for PTZ to act upon. As with the mood stabilizers, the effects of CBZ and PTZ were both drug- and genotype-dependent.

Finally, our findings that MPEP made WT behave like *shank3ab-/-* larvae in the VMR assay suggest that blocking mGluR5 may affect sensory processing. MPEP blocks mGluR5 and improves excessive grooming and striatal synaptic plasticity in a mouse *shank3* model ( Wang *et al*., 2016). GluR5 continues to show promise as a regulator of excitatory/inhibitory balance in the striatum where a negative correlation between mGluR5 and GABA was measured in autistic people using fMRI; mouse *Cntnap2* mutants showed a similar negative mGluR5 and GABA correlation that was not found in either *Shank3* or *16p11.2* deletion models ( Carey *et al*., 2022).

## Summary/conclusions

Our findings highlight the genotype-, drug-, and phenotype-specific challenges of designing treatment strategies for Phelan-McDermid Syndrome. These include trade-offs that can occur when a drug like risperidone improves sensory-processing and mood at the expense of gut function and differential effects of drugs on different symptoms.

**Table 2.** ANOVA of 30-second transition from lights-on to lights-off for DMSO-exposed WT, *shank3abN-/-*, and *shank3abC-/-* larvae. See Figure 1c.

Table analyzed	DMSO 30sec paired				
Two-way ANOVA	Ordinary				
Alpha	0.05				
Source of Variation	% of total variation	P value	P value summary	Significant?	
Interaction	4.261	0.0017	**	Yes	
Row Factor Light	5.139	0.0005	***	Yes	
Column Factor genotype	17.21	<0.0001	****	Yes	
ANOVA table	SS (Type III)	DF	MS	F (DFn, DFd)	P value
Interaction	4421	2	2211	F (2, 214) = 6.588	P=0.0017
Row Factor genotype	5333	2	2666	F (2, 214) = 7.946	P=0.0005
Column Factor light	17854	1	17854	F (1, 214) = 53.20	P<0.0001
Residual	71814	214	335.6		
Difference between column means					
Predicted (LS) mean of Lights-on	21.82				
Predicted (LS) mean of Lights-off	40.23				
Difference between predicted means	-18.41				
SE of difference	2.523				
95% CI of difference	-23.38 to -13.43				
Data summary					
Number of columns (Light)	2				
Number of rows (genotype)	3				
Number of values	220				

* p < 0.05,

** p < 0.01,

*** p < 0.001,

**** p < 0.0001.

**Table 3.** Paired comparisons of 30-second transition from lights-on to lights-off for DMSO-exposed WT, *shank3abN-/-*, and *shank3abC-/-*larvae. See Figure 1c.

Paired Comparison Lights-on to Lights-off								
Number of families	1							
Number of comparisons per family	3							
Alpha	0.05							
Bonferroni's multiple comparisons test	Predicted (LS) mean diff.	95.00% CI of diff.	Below threshold?	Summary	Adjusted P Value			
Lights-on - Lights-off								
WT	-30.24	-39.46 to -21.02	Yes	****	<0.0001			
*shank3abN-/-*	-9.882	-20.93 to 1.169	No	ns	0.0962			
*shank3abC-/-*	-15.09	-26.34 to -3.843	Yes	**	0.0042			
Test details	Predicted (LS) mean 1	Predicted (LS) mean 2	Predicted (LS) mean diff.	SE of diff.	N1	N2	t	DF
Lights-on - Lights-off								
WT	22.31	52.55	-30.24	3.82	46	46	7.917	214
*shank3abN-/-*	21.11	30.99	-9.882	4.58	32	32	2.158	214
*shank3abC-/-*	22.04	37.13	-15.09	4.662	38	26	3.237	214

* p < 0.05,

** p < 0.01,

*** p < 0.001,

**** p < 0.0001.

**Table 4.** ANOVA of 30-second lights-off for DMSO-exposed WT, *shank3abN-/-*, and *shank3abC-/-* larvae. See Figure 1d.

Table analyzed	First 30 sec Off
Kruskal-Wallis test	
P value	<0.001
Exact or approximate P value?	Approximate
P value summary	***
Do the medians vary signif. (P < 0.05)?	Yes
Number of groups	3
Kruskal-Wallis statistic	54.04
Data summary	
Number of treatments (columns)	3
Number of values (total)	159

* p < 0.05,

** p < 0.01,

*** p < 0.001,

**** p < 0.0001.

**Table 5.** Dunn’s multiple comparisons of 30-second transition from lights-on to lights-off for DMSO-exposed WT, *shank3abN-/-*, and *shank3abC-/-* larvae. See Figure 1d.

Number of families	1					
Number of comparisons per family	3					
Alpha	0.05					
Dunn's multiple comparisons test	Mean rank diff.	Significant?	Summary	Adjusted P Value		
WT vs. *shank3abN-/-*	61.85	Yes	***	<0.001	A-B	
WT vs. *shank3abC-/-*	49.2	Yes	***	<0.001	A-C	
*shank3abN* vs. *shank3abC*	-12.66	No	ns	0.48	B-C	
Test details	Mean rank 1	Mean rank 2	Mean rank diff.	n1	n2	Z
WT vs. *shank3abN-/-*	118.9	57.05	61.85	50	65	7.142
WT vs. *shank3abC-/-*	118.9	69.7	49.2	50	44	5.169
*shank3abN* vs. *shank3abC*	57.05	69.7	-12.66	65	44	1.408

* p < 0.05,

** p < 0.01,

*** p < 0.001,

**** p < 0.0001.

**Table 6.** ANOVA of 5-minute lights-off for DMSO-exposed WT, *shank3abN-/-*, and *shank3abC-/-*larvae. See Figure 1e.

Table analyzed	5 min Off
Kruskal-Wallis test	
P value	<0.001
Exact or approximate P value?	Approximate
P value summary	***
Do the medians vary signif. (P < 0.05)?	Yes
Number of groups	3
Kruskal-Wallis statistic	40.54
Data summary	
Number of treatments (columns)	3
Number of values (total)	159

* p < 0.05,

** p < 0.01,

*** p < 0.001,

**** p < 0.0001.

**Table 7.** Dunn’s multiple comparisons of 5-minute transition from lights-on to lights-off for DMSO-exposed WT, *shank3abN-/-*, and *shank3abC-/-*larvae. See Figure 1e.

Number of families	1					
Number of comparisons per family	3					
Alpha	0.05					
Dunn's multiple comparisons test	Mean rank diff.	Significant?	Summary	Adjusted P Value		
WT vs. *shank3abN-/-*	53.78	Yes	***	<0.001	A-B	
WT vs. *shank3abC-/-*	41.9	Yes	***	<0.001	A-C	
*shank3abN* vs. *shank3abC*	-11.88	No	ns	0.56	B-C	
Test details	Mean rank 1	Mean rank 2	Mean rank diff.	n1	n2	Z
WT vs. *shank3abN-/-*	113.6	59.8	53.78	50	65	6.209
WT vs. *shank3abC-/-*	113.6	71.68	41.9	50	44	4.402
*shank3abN* vs. *shank3abC*	59.8	71.68	-11.88	65	44	1.322

* p < 0.05,

** p < 0.01,

*** p < 0.001,

**** p < 0.0001.

**Table 8.** ANOVA of 30-second light-off for risperidone dose response curve in WT larvae. See Figure 2a.

Table analyzed	Wildtype risperidone dose response 30 sec Lights-off
Kruskal-Wallis test	
P value	<0.0001
Exact or approximate P value?	Approximate
P value summary	****
Do the medians vary signif. (P < 0.05)?	Yes
Number of groups [Risperidone]	4
Kruskal-Wallis statistic	24.09
Data summary	
Number of treatments (columns)	4
Number of values (total)	114

* p < 0.05,

** p < 0.01,

*** p < 0.001,

**** p < 0.0001.

**Table 9.** Dunn’s multiple comparisons of 30-second lights-off for risperidone dose response curve. See Figure 2a.

Number of families	1					
Number of comparisons per family	6					
Alpha	0.05					
Dunn's multiple comparisons test	Mean rank diff.	Significant?	Summary	Adjusted P Value		
WT vs. 1 uM Risp	10.89	No	ns	>0.9999	A-B	
WT vs. 10 uM RIsp	26.1	Yes	**	0.0015	A-C	
WT vs. 20 uM Risp	48.19	Yes	***	0.0003	A-D	
1 uM Risp vs. 10 uM RIsp	15.22	No	ns	0.753	B-C	
1 uM Risp vs. 20 uM Risp	37.3	Yes	*	0.0406	B-D	
10 uM RIsp vs. 20 uM Risp	22.08	No	ns	0.4381	C-D	
Test details	Mean rank 1	Mean rank 2	Mean rank diff.	n1	n2	Z
WT vs. 1 uM Risp	71.08	60.19	10.89	53	16	1.155
WT vs. 10 uM RIsp	71.08	44.97	26.1	53	36	3.657
WT vs. 20 uM Risp	71.08	22.89	48.19	53	9	4.044
1 uM Risp vs. 10 uM RIsp	60.19	44.97	15.22	16	36	1.532
1 uM Risp vs. 20 uM Risp	60.19	22.89	37.3	16	9	2.708
10 uM RIsp vs. 20 uM Risp	44.97	22.89	22.08	36	9	1.793

* p < 0.05,

** p < 0.01,

*** p < 0.001,

**** p < 0.0001.

**Table 10.** ANOVA of 5-minute lights-off for risperidone dose response curve. See Figure 2a.

Table analyzed	Wildtype risperidone dose response 5 min Lights-off
Kruskal-Wallis test	
P value	<0.0001
Exact or approximate P value?	Approximate
P value summary	****
Do the medians vary signif. (P < 0.05)?	Yes
Number of groups [risperidone]	4
Kruskal-Wallis statistic	24.89
Data summary	
Number of treatments (columns)	4
Number of values (total)	115

* p < 0.05,

** p < 0.01,

*** p < 0.001,

**** p < 0.0001.

**Table 11.** Dunn’s multiple comparisons of 5-minute lights-off for risperidone dose response curve. See Figure 2a.

Number of families	1					
Number of comparisons per family	6					
Alpha	0.05					
Dunn's multiple comparisons test	Mean rank diff.	Significant?	Summary	Adjusted P Value		
WT vs. 1 uM	3.684	No	ns	>0.9999	A-B	
WT vs. 10 uM	16.41	No	ns	0.1362	A-C	
WT vs. 20 uM	55.03	Yes	****	<0.0001	A-D	
1 uM vs. 10 uM	12.72	No	ns	>0.9999	B-C	
1 uM vs. 20 uM	51.35	Yes	***	0.0008	B-D	
10 uM vs. 20 uM	38.63	Yes	**	0.0071	C-D	
Test details	Mean rank 1	Mean rank 2	Mean rank diff.	n1	n2	Z
WT vs. 1 uM	68.43	64.75	3.684	53	16	0.3873
WT vs. 10 uM	68.43	52.03	16.41	53	36	2.278
WT vs. 20 uM	68.43	13.4	55.03	53	10	4.788
1 uM vs. 10 uM	64.75	52.03	12.72	16	36	1.27
1 uM vs. 20 uM	64.75	13.4	51.35	16	10	3.821
10 uM vs. 20 uM	52.03	13.4	38.63	36	10	3.241

* p < 0.05,

** p < 0.01,

*** p < 0.001,

**** p < 0.0001.

**Table 12.** ANOVA of 30-second light-off for lithium chloride dose response curve. See Figure 2b.

Table analyzed	Wildtype LiCL dose response 30 sec Lights-off
Kruskal-Wallis test	
P value	0.2423
Exact or approximate P value?	Approximate
P value summary	ns
Do the medians vary signif. (P < 0.05)?	No
Number of groups [LiCl]	4
Kruskal-Wallis statistic	4.184
Data summary	
Number of treatments (columns)	4
Number of values (total)	55

**Table 13.** ANOVA of 5-minute lights-off for lithium chloride dose response curve. See Figure 2b.

Table analyzed	Wildtype LiCL dose response 5 min Lights-off
Kruskal-Wallis test	
P value	0.9904
Exact or approximate P value?	Approximate
P value summary	ns
Do the medians vary signif. (P < 0.05)?	No
Number of groups [LiCl]	4
Kruskal-Wallis statistic	0.1118
Data summary	
Number of treatments (columns)	4
Number of values (total)	55

**Table 14.** ANOVA of 30-second light-off for carbamazepine dose response curve. See Figure 2c.

Table analyzed	Wildtype CBZ dose response 30 sec Lights-off
Kruskal-Wallis test	
P value	0.0422
Exact or approximate P value?	Approximate
P value summary	*
Do the medians vary signif. (P < 0.05)?	Yes
Number of groups [CBZ]	4
Kruskal-Wallis statistic	8.191
Data summary	
Number of treatments (columns)	4
Number of values (total)	97

* p < 0.05,

** p < 0.01,

*** p < 0.001,

**** p < 0.0001.

**Table 15.** Dunn’s multiple comparisons of 30-second lights-off for carbamazepine dose response curve. See Figure 2c.

Number of families	1					
Number of comparisons per family	3					
Alpha	0.05					
Dunn's multiple comparisons test	Mean rank diff.	Significant?	Summary	Adjusted P Value	A-?	
WT vs. WT 80 uM CBZ	8.93	No	ns	0.8191	B	WT CBZ
WT vs. WT 120 uM CBZ	1.646	No	ns	>0.9999	C	WT CBZ 2
WT vs. WT CBZ 200 uM CBZ	22	Yes	*	0.0172	D	WT CBZ 3
Test details	Mean rank 1	Mean rank 2	Mean rank diff.	n1	n2	Z
WT vs. WT 80 uM CBZ CBZ	54.62	45.69	8.93	47	16	1.096
WT vs. WT 120 uM CBZ CBZ	54.62	52.97	1.646	47	17	0.2067
WT vs. WT 200 uM CBZ CBZ	54.62	32.62	22	47	17	2.762

* p < 0.05,

** p < 0.01,

*** p < 0.001,

**** p < 0.0001.

**Table 16.** ANOVA of 5-minute lights-off for carbamazepine dose response curve. See Figure 2c.

Table analyzed	Wildtype CBZ 5 min Lights-off
Kruskal-Wallis test	
P value	<0.0001
Exact or approximate P value?	Approximate
P value summary	****
Do the medians vary signif. (P < 0.05)?	Yes
Number of groups	4
Kruskal-Wallis statistic	28.11
Data summary	
Number of treatments (columns)	4
Number of values (total)	97

* p < 0.05,

** p < 0.01,

*** p < 0.001,

**** p < 0.0001.

**Table 17.** Dunn’s multiple comparisons of 5-minute lights-off for carbamazepine dose response curve. See Figure 2c.

Number of families	1					
Number of comparisons per family	3					
Alpha	0.05					
Dunn's multiple comparisons test	Mean rank diff.	Significant?	Summary	Adjusted P Value	A-?	
WT vs. WT 80 uM CBZ	30.76	Yes	***	0.0005	B	WT CBZ
WT vs. WT 120 uM CBZ CBZ	32.83	Yes	***	0.0001	C	WT CBZ
WT vs. 20 uM CBZ WT cbz	26.72	Yes	**	0.0024	D	WT cbz
Test details	Mean rank 1	Mean rank 2	Mean rank diff.	n1	n2	Z
WT vs. WT 80 uM CBZ	64.51	33.75	30.76	47	16	3.776
WT vs. WT 120 uM CBZ	64.51	31.68	32.83	47	17	4.122
WT vs. WT 200 uM CBZ	64.51	37.79	26.72	47	17	3.354

* p < 0.05,

** p < 0.01,

*** p < 0.001,

**** p < 0.0001.

**Table 18.** ANOVA of 30-second lights-off for MPEP dose response curve. See Figure 2d.

Table analyzed	Wildtype MPEP 30 sec Lights-off
Kruskal-Wallis test	
P value	<0.0001
Exact or approximate P value?	Approximate
P value summary	****
Do the medians vary signif. (P < 0.05)?	Yes
Number of groups	4
Kruskal-Wallis statistic	50.85
Data summary	
Number of treatments (columns)	4
Number of values (total)	68

* p < 0.05,

** p < 0.01,

*** p < 0.001,

**** p < 0.0001.

**Table 19.** Dunn’s multiple comparisons of 30-second lights-off for MPEP dose response curve. See Figure 2d.

Number of families	1					
Number of comparisons per family	3					
Alpha	0.05					
Dunn’s multiple comparisons test	Mean rank diff.	Significant?	Summary	Adjusted P Value	A-?	
WT vs. WT 1 uM MPEP	12.47	No	ns	0.1319	B	WT 1 uM MPEP
WT vs. WT 5 uM MPEP	33.87	Yes	****	<0.0001	C	WT 5 uM MPEP
WT vs. WT 10 uM MPEP	41.34	Yes	****	<0.0001	D	WT 10 uM MPEP
Test details	Mean rank 1	Mean rank 2	Mean rank diff.	n1	n2	Z
WT vs. WT 1 uM MPEP	51.04	38.56	12.47	28	16	2.014
WT vs. WT 5 uM MPEP	51.04	17.17	33.87	28	9	4.474
WT vs. WT 10 uM MPEP	51.04	9.7	41.34	28	15	6.538

* p < 0.05,

** p < 0.01,

*** p < 0.001,

**** p < 0.0001.

**Table 20.** ANOVA of 5-minute lights-off for MPEP dose response curve. See Figure 2d.

Table analyzed	Wildtype MPEP dose response 5 min Lights-off
Kruskal-Wallis test	
P value	<0.0001
Exact or approximate P value?	Approximate
P value summary	****
Do the medians vary signif. (P < 0.05)?	Yes
Number of groups	4
Kruskal-Wallis statistic	50.52
Data summary	
Number of treatments (columns)	4
Number of values (total)	66

* p < 0.05,

** p < 0.01,

*** p < 0.001,

**** p < 0.0001.

**Table 21.** Dunn’s multiple comparisons of 5-minute lights-off for MPEP dose response curve. See Figure 2d.

Number of families	1					
Number of comparisons per family	3					
Alpha	0.05					
Dunn's multiple comparisons test	Mean rank diff.	Significant?	Summary	Adjusted P Value	A-?	
WT vs. WT 1 μM MPEP	13.96	No	ns	0.0788	B	WT 1 uM MPEP
WT vs. WT 5 μM MPEP	31.48	Yes	****	<0.0001	C	WT 5 uM MPEP
WT vs. WT 10 μM MPEP	40.84	Yes	****	<0.0001	D	WT 10 uM MPEP
Test details	Mean rank 1	Mean rank 2	Mean rank diff.	n1	n2	Z
WT vs. WT 1 μM MPEP	50.04	36.07	13.96	28	14	2.222
WT vs. WT 5 μM MPEP	50.04	18.56	31.48	28	9	4.28
WT vs. WT 10 μM MPEP	50.04	9.2	40.84	28	15	6.649

* p < 0.05,

** p < 0.01,

*** p < 0.001,

**** p < 0.0001.

**Table 22.** ANOVA of 30-second light-off for 10 μM risperidone-exposed WT, *shank3abN-/-*, and *shank3abC-/-* larvae. See Figure 3a.

Table analyzed	risperidone 30 sec lights-off
Kruskal-Wallis test	
P value	<0.0001
Exact or approximate P value?	Approximate
P value summary	****
Do the medians vary signif. (P < 0.05)?	Yes
Number of groups	6
Kruskal-Wallis statistic	74.44
Data summary	
Number of treatments (columns)	6
Number of values (total)	155

* p < 0.05,

** p < 0.01,

*** p < 0.001,

**** p < 0.0001.

**Table 23.** Dunn’s multiple comparisons of 30-second lights-off for 10 μM risperidone-exposed WT, *shank3abN-/-*, and *shank3abC-/-* larvae. See Figure 3a.

Number of families	1					
Number of comparisons per family	15					
Alpha	0.05					
Dunn's multiple comparisons test	Mean rank diff.	Significant?	Summary	Adjusted P Value		
WT dmso vs. WT risp	4.607	No	ns	>0.9999	A-B	
WT dmso vs. shk3n dmso	50.65	Yes	***	0.0003	A-C	
WT dmso vs. shk3n risp	78.7	Yes	****	<0.0001	A-D	
WT dmso vs. shk3c dmso	41.9	Yes	**	0.0059	A-E	
WT dmso vs. shk3c risp	70.75	Yes	****	<0.0001	A-F	
WT risp vs. shk3n dmso	37.29	Yes	*	0.0284	B-C	
WT risp vs. shk3n risp	74.1	Yes	****	<0.0001	B-D	
WT risp vs. shk3c dmso	66.14	Yes	****	<0.0001	B-E	
WT risp vs. shk3c risp	46.05	Yes	**	0.0022	B-F	
shk3n dmso vs. shk3n risp	28.05	No	ns	0.4389	C-D	
shk3n dmso vs. shk3c dmso	-8.756	No	ns	>0.9999	C-E	
shk3n dmso vs. shk3c risp	20.1	No	ns	>0.9999	C-F	
shk3n risp vs. shk3c dmso	-36.81	No	ns	0.0578	D-E	
shk3n risp vs. shk3c risp	-7.955	No	ns	>0.9999	D-F	
shk3c dmso vs. shk3c risp	28.85	No	ns	0.3522	E-F	
Test details	Mean rank 1	Mean rank 2	Mean rank diff.	n1	n2	Z
WT dmso vs. WT risp	112.5	107.9	4.607	31	29	0.4063
WT dmso vs. shk3n dmso	112.5	61.85	50.65	31	24	4.235
WT dmso vs. shk3n risp	112.5	33.8	78.7	31	23	6.498
WT dmso vs. shk3c dmso	112.5	70.6	41.9	31	25	3.545
WT dmso vs. shk3c risp	112.5	41.75	70.75	31	23	5.841
WT risp vs. shk3n dmso	107.9	61.85	46.05	29	24	3.792
WT risp vs. shk3n risp	107.9	33.8	74.1	29	23	6.027
WT risp vs. shk3c dmso	107.9	70.6	37.29	29	25	3.107
WT risp vs. shk3c risp	107.9	41.75	66.14	29	23	5.38
shk3n dmso vs. shk3n risp	61.85	33.8	28.05	24	23	2.18
shk3n dmso vs. shk3c dmso	61.85	70.6	-8.756	24	25	0.6954
shk3n dmso vs. shk3c risp	61.85	41.75	20.1	24	23	1.562
shk3n risp vs. shk3c dmso	33.8	70.6	-36.81	23	25	2.89
shk3n risp vs. shk3c risp	33.8	41.75	-7.955	23	23	0.6114
shk3c dmso vs. shk3c risp	70.6	41.75	28.85	25	23	2.266

* p < 0.05,

** p < 0.01,

*** p < 0.001,

**** p < 0.0001.

**Table 24.** ANOVA of 5-minute lights-off for 10 μM risperidone-exposed WT, *shank3abN-/-*, and *shank3abC-/-* larvae. See Figure 3b.

Table analyzed	risperidone 5 min lights-off
Kruskal-Wallis test	
P value	<0.0001
Exact or approximate P value?	Approximate
P value summary	****
Do the medians vary signif. (P < 0.05)?	Yes
Number of groups	6
Kruskal-Wallis statistic	27.87
Data summary	
Number of treatments (columns)	6
Number of values (total)	155

* p < 0.05,

** p < 0.01,

*** p < 0.001,

**** p < 0.0001.

**Table 25.** Dunn’s multiple comparisons of 5-minute lights-off for 10 μM risperidone-exposed WT, *shank3abN-/-*, and *shank3abC-/-* larvae. See Figure 3b.

Number of families	1					
Number of comparisons per family	15					
Alpha	0.05					
Dunn's multiple comparisons test	Mean rank diff.	Significant?	Summary	Adjusted P Value		
WT dmso vs. WT risp	0.9774	No	ns	>0.9999	A-B	
WT dmso vs. shk3n dmso	45.1	Yes	**	0.0024	A-C	
WT dmso vs. shk3n risp	32.82	No	ns	0.1012	A-D	
WT dmso vs. shk3c dmso	39.86	Yes	*	0.0112	A-E	
WT dmso vs. shk3c risp	33.23	No	ns	0.0914	A-F	
WT risp vs. shk3n dmso	44.12	Yes	**	0.0042	B-C	
WT risp vs. shk3n risp	31.84	No	ns	0.1442	B-D	
WT risp vs. shk3c dmso	38.88	Yes	*	0.018	B-E	
WT risp vs. shk3c risp	32.25	No	ns	0.1308	B-F	
shk3n dmso vs. shk3n risp	-12.28	No	ns	>0.9999	C-D	
shk3n dmso vs. shk3c dmso	-5.241	No	ns	>0.9999	C-E	
shk3n dmso vs. shk3c risp	-11.87	No	ns	>0.9999	C-F	
shk3n risp vs. shk3c dmso	7.042	No	ns	>0.9999	D-E	
shk3n risp vs. shk3c risp	0.4091	No	ns	>0.9999	D-F	
shk3c dmso vs. shk3c risp	-6.633	No	ns	>0.9999	E-F	
Test details	Mean rank 1	Mean rank 2	Mean rank diff.	n1	n2	Z
WT dmso vs. WT risp	98.32	97.34	0.9774	31	29	0.08619
WT dmso vs. shk3n dmso	98.32	53.22	45.1	31	24	3.771
WT dmso vs. shk3n risp	98.32	65.5	32.82	31	23	2.709
WT dmso vs. shk3c dmso	98.32	58.46	39.86	31	25	3.372
WT dmso vs. shk3c risp	98.32	65.09	33.23	31	23	2.743
WT risp vs. shk3n dmso	97.34	53.22	44.12	29	24	3.633
WT risp vs. shk3n risp	97.34	65.5	31.84	29	23	2.59
WT risp vs. shk3c dmso	97.34	58.46	38.88	29	25	3.239
WT risp vs. shk3c risp	97.34	65.09	32.25	29	23	2.623
shk3n dmso vs. shk3n risp	53.22	65.5	-12.28	24	23	0.9544
shk3n dmso vs. shk3c dmso	53.22	58.46	-5.241	24	25	0.4162
shk3n dmso vs. shk3c risp	53.22	65.09	-11.87	24	23	0.9226
shk3n risp vs. shk3c dmso	65.5	58.46	7.042	23	25	0.5528
shk3n risp vs. shk3c risp	65.5	65.09	0.4091	23	23	0.03144
shk3c dmso vs. shk3c risp	58.46	65.09	-6.633	25	23	0.5207

* p < 0.05,

** p < 0.01,

*** p < 0.001,

**** p < 0.0001.

**Table 26.** ANOVA of 30-second light-off for 5 mM lithium chloride-exposed WT, *shank3abN-/-*, and *shank3abC-/-* larvae. See Figure 4a.

Table analyzed	LiCL 30 sec lights-off
Kruskal-Wallis test	
P value	<0.0001
Exact or approximate P value?	Approximate
P value summary	****
Do the medians vary signif. (P < 0.05)?	Yes
Number of groups	6
Kruskal-Wallis statistic	60.74
Data summary	
Number of treatments (columns)	6
Number of values (total)	104

* p < 0.05,

** p < 0.01,

*** p < 0.001,

**** p < 0.0001.

**Table 27.** Dunn’s multiple comparisons of 30-second lights-off for 5 mM lithium chloride-exposed WT, *shank3abN-/-*, and *shank3abC-/-* larvae. See Figure 4a.

Number of families	1					
Number of comparisons per family	15					
Alpha	0.05					
Dunn's multiple comparisons test	Mean rank diff.	Significant?	Summary	Adjusted P Value		
WT vs. WT LiCL	-5.725	No	ns	>0.9999	A-B	
WT vs. shk3n hom	46.9	Yes	****	<0.0001	A-C	
WT vs. shk3n hom LiCL	42.23	Yes	***	0.0002	A-D	
WT vs. shk3c hom	49.59	Yes	****	<0.0001	A-E	
WT vs. shk3c hom LiCL	43.71	Yes	***	0.0002	A-F	
WT LiCL vs. shk3n hom	52.63	Yes	****	<0.0001	B-C	
WT LiCL vs. shk3n hom LiCL	47.96	Yes	****	<0.0001	B-D	
WT LiCL vs. shk3c hom	55.31	Yes	****	<0.0001	B-E	
WT LiCL vs. shk3c hom LiCL	49.44	Yes	****	<0.0001	B-F	
shk3n hom vs. shk3n hom LiCL	-4.667	No	ns	>0.9999	C-D	
shk3n hom vs. shk3c hom	2.688	No	ns	>0.9999	C-E	
shk3n hom vs. shk3c hom LiCL	-3.188	No	ns	>0.9999	C-F	
shk3n hom LiCL vs. shk3c hom	7.354	No	ns	>0.9999	D-E	
shk3n hom LiCL vs. shk3c hom LiCL	1.479	No	ns	>0.9999	D-F	
shk3c hom vs. shk3c hom LiCL	-5.875	No	ns	>0.9999	E-F	
Test details	Mean rank 1	Mean rank 2	Mean rank diff.	n1	n2	Z
WT vs. WT LiCL	81.4	87.13	-5.725	20	16	0.5658
WT vs. shk3n hom	81.4	34.5	46.9	20	18	4.785
WT vs. shk3n hom LiCL	81.4	39.17	42.23	20	18	4.309
WT vs. shk3c hom	81.4	31.81	49.59	20	16	4.901
WT vs. shk3c hom LiCL	81.4	37.69	43.71	20	16	4.32
WT LiCL vs. shk3n hom	87.13	34.5	52.63	16	18	5.077
WT LiCL vs. shk3n hom LiCL	87.13	39.17	47.96	16	18	4.627
WT LiCL vs. shk3c hom	87.13	31.81	55.31	16	16	5.186
WT LiCL vs. shk3c hom LiCL	87.13	37.69	49.44	16	16	4.635
shk3n hom vs. shk3n hom LiCL	34.5	39.17	-4.667	18	18	0.4641
shk3n hom vs. shk3c hom	34.5	31.81	2.688	18	16	0.2593
shk3n hom vs. shk3c hom LiCL	34.5	37.69	-3.188	18	16	0.3075
shk3n hom LiCL vs. shk3c hom	39.17	31.81	7.354	18	16	0.7095
shk3n hom LiCL vs. shk3c hom LiCL	39.17	37.69	1.479	18	16	0.1427
shk3c hom vs. shk3c hom LiCL	31.81	37.69	-5.875	16	16	0.5508

* p < 0.05,

** p < 0.01,

*** p < 0.001,

**** p < 0.0001.

**Table 28.** ANOVA of 5-minute lights-off for 5 mM lithium chloride-exposed WT, *shank3abN-/-*, and *shank3abC-/-* larvae. See Figure 5b.

Table analyzed	LiCL 5 min lights-off
Kruskal-Wallis test	
P value	<0.0001
Exact or approximate P value?	Approximate
P value summary	****
Do the medians vary signif. (P < 0.05)?	Yes
Number of groups	6
Kruskal-Wallis statistic	49.22
Data summary	
Number of treatments (columns)	6
Number of values (total)	104

* p < 0.05,

** p < 0.01,

*** p < 0.001,

**** p < 0.0001.

**Table 29.** Dunn’s multiple comparisons of 5-minute lights-off for 5 mM lithium chloride-exposed WT, *shank3abN-/-*, and *shank3abC-/-* larvae. See Figure 5b.

Number of families	1					
Number of comparisons per family	15					
Alpha	0.05					
Dunn's multiple comparisons test	Mean rank diff.	Significant?	Summary	Adjusted P Value		
WT vs. WT LiCL	9.069	No	ns	>0.9999	A-B	
WT vs. shk3n hom	55.96	Yes	****	<0.0001	A-C	
WT vs. shk3n hom LiCL	46.02	Yes	****	<0.0001	A-D	
WT vs. shk3c hom	39.07	Yes	**	0.0017	A-E	
WT vs. shk3c hom LiCL	40.91	Yes	***	0.0008	A-F	
WT LiCL vs. shk3n hom	46.89	Yes	****	<0.0001	B-C	
WT LiCL vs. shk3n hom LiCL	36.95	Yes	**	0.0055	B-D	
WT LiCL vs. shk3c hom	30	No	ns	0.0737	B-E	
WT LiCL vs. shk3c hom LiCL	31.84	Yes	*	0.0424	B-F	
shk3n hom vs. shk3n hom LiCL	-9.944	No	ns	>0.9999	C-D	
shk3n hom vs. shk3c hom	-16.89	No	ns	>0.9999	C-E	
shk3n hom vs. shk3c hom LiCL	-15.05	No	ns	>0.9999	C-F	
shk3n hom LiCL vs. shk3c hom	-6.948	No	ns	>0.9999	D-E	
shk3n hom LiCL vs. shk3c hom LiCL	-5.104	No	ns	>0.9999	D-F	
shk3c hom vs. shk3c hom LiCL	1.844	No	ns	>0.9999	E-F	
Test details	Mean rank 1	Mean rank 2	Mean rank diff.	n1	n2	Z
WT vs. WT LiCL	83.85	74.78	9.069	20	16	0.8963
WT vs. shk3n hom	83.85	27.89	55.96	20	18	5.71
WT vs. shk3n hom LiCL	83.85	37.83	46.02	20	18	4.695
WT vs. shk3c hom	83.85	44.78	39.07	20	16	3.861
WT vs. shk3c hom LiCL	83.85	42.94	40.91	20	16	4.044
WT LiCL vs. shk3n hom	74.78	27.89	46.89	16	18	4.524
WT LiCL vs. shk3n hom LiCL	74.78	37.83	36.95	16	18	3.565
WT LiCL vs. shk3c hom	74.78	44.78	30	16	16	2.813
WT LiCL vs. shk3c hom LiCL	74.78	42.94	31.84	16	16	2.986
shk3n hom vs. shk3n hom LiCL	27.89	37.83	-9.944	18	18	0.989
shk3n hom vs. shk3c hom	27.89	44.78	-16.89	18	16	1.63
shk3n hom vs. shk3c hom LiCL	27.89	42.94	-15.05	18	16	1.452
shk3n hom LiCL vs. shk3c hom	37.83	44.78	-6.948	18	16	0.6703
shk3n hom LiCL vs. shk3c hom LiCL	37.83	42.94	-5.104	18	16	0.4924
shk3c hom vs. shk3c hom LiCL	44.78	42.94	1.844	16	16	0.1729

* p < 0.05,

** p < 0.01,

*** p < 0.001,

**** p < 0.0001.

**Table 30.** ANOVA of 30-second light-off for 200 μM carbamazepine exposed *shank3ab* larvae. See Figure 5a.

Table analyzed	CBZ 30 sec lights-off
Kruskal-Wallis test	
P value	<0.0001
Exact or approximate P value?	Approximate
P value summary	****
Do the medians vary signif. (P < 0.05)?	Yes
Number of groups	6
Kruskal-Wallis statistic	73.6
Data summary	
Number of treatments (columns)	6
Number of values (total)	176

* p < 0.05,

** p < 0.01,

*** p < 0.001,

**** p < 0.0001.

**Table 31.** Dunn’s multiple comparisons of 30-second lights-off for 200 μM carbamazepine-exposed WT, *shank3abN-/-*, and *shank3abC-/-* larvae. See Figure 5a.

Number of families	1					
Number of comparisons per family	15					
Alpha	0.05					
Dunn's multiple comparisons test	Mean rank diff.	Significant?	Summary	Adjusted P Value		
WT vs. WT CBZ	14.04	No	ns	>0.9999	A-B	
WT vs. shk3n	61.73	Yes	****	<0.0001	A-C	
WT vs. shk3n CBZ	112.4	Yes	****	<0.0001	A-D	
WT vs. shk3c	42.38	Yes	*	0.0175	A-E	
WT vs. shk3c CBZ	39.99	Yes	*	0.0167	A-F	
WT CBZ vs. shk3n	47.69	Yes	**	0.0053	B-C	
WT CBZ vs. shk3n CBZ	98.35	Yes	****	<0.0001	B-D	
WT CBZ vs. shk3c	28.34	No	ns	0.4199	B-E	
WT CBZ vs. shk3c CBZ	25.95	No	ns	0.4803	B-F	
shk3n vs. shk3n CBZ	50.66	Yes	*	0.0181	C-D	
shk3n vs. shk3c	-19.35	No	ns	>0.9999	C-E	
shk3n vs. shk3c CBZ	-21.74	No	ns	>0.9999	C-F	
shk3n CBZ vs. shk3c	-70.01	Yes	****	<0.0001	D-E	
shk3n CBZ vs. shk3c CBZ	-72.39	Yes	****	<0.0001	D-F	
shk3c vs. shk3c CBZ	-2.383	No	ns	>0.9999	E-F	
Test details	Mean rank 1	Mean rank 2	Mean rank diff.	n1	n2	Z
WT vs. WT CBZ	126.2	112.2	14.04	35	37	1.169
WT vs. shk3n	126.2	64.5	61.73	35	24	4.571
WT vs. shk3n CBZ	126.2	13.84	112.4	35	19	7.741
WT vs. shk3c	126.2	83.85	42.38	35	27	3.247
WT vs. shk3c CBZ	126.2	86.24	39.99	35	34	3.26
WT CBZ vs. shk3n	112.2	64.5	47.69	37	24	3.571
WT CBZ vs. shk3n CBZ	112.2	13.84	98.35	37	19	6.839
WT CBZ vs. shk3c	112.2	83.85	28.34	37	27	2.197
WT CBZ vs. shk3c CBZ	112.2	86.24	25.95	37	34	2.144
shk3n vs. shk3n CBZ	64.5	13.84	50.66	24	19	3.238
shk3n vs. shk3c	64.5	83.85	-19.35	24	27	1.354
shk3n vs. shk3c CBZ	64.5	86.24	-21.74	24	34	1.6
shk3n CBZ vs. shk3c	13.84	83.85	-70.01	19	27	4.589
shk3n CBZ vs. shk3c CBZ	13.84	86.24	-72.39	19	34	4.96
shk3c vs. shk3c CBZ	83.85	86.24	-2.383	27	34	0.1815

* p < 0.05,

** p < 0.01,

*** p < 0.001,

**** p < 0.0001.

**Table 32.** ANOVA of 5-minute lights-off for 200 μM carbamazepine-exposed WT, *shank3abN-/-*, and *shank3abC-/-* larvae. See Figure 5b.

Table analyzed	CBZ 5 min lights-off
Kruskal-Wallis test	
P value	<0.0001
Exact or approximate P value?	Approximate
P value summary	****
Do the medians vary signif. (P < 0.05)?	Yes
Number of groups	6
Kruskal-Wallis statistic	40.2
Data summary	
Number of treatments (columns)	6
Number of values (total)	176

* p < 0.05,

** p < 0.01,

*** p < 0.001,

**** p < 0.0001.

**Table 33.** Dunn’s multiple comparisons of 5-minute lights-off for 200 μM carbamazepine-exposed WT, *shank3abN-/-*, and *shank3abC-/-* larvae. See Figure 5b.

Number of families	1					
Number of comparisons per family	15					
Alpha	0.05					
Dunn's multiple comparisons test	Mean rank diff.	Significant?	Summary	Adjusted P Value		
WT vs. WT cbz	25.72	No	ns	0.4844	A-B	
WT vs. shank3n	62.12	Yes	***	0.0003	A-C	
WT vs. shank3n CBZ	72.55	Yes	****	<0.0001	A-D	
WT vs. shank3c	51.91	Yes	**	0.001	A-E	
WT vs. shank3c CBZ	48.3	Yes	**	0.0012	A-F	
WT cbz vs. shank3n	36.4	No	ns	0.1706	B-C	
WT cbz vs. shank3n CBZ	46.83	Yes	**	0.0068	B-D	
WT cbz vs. shank3c	26.19	No	ns	0.6338	B-E	
WT cbz vs. shank3c CBZ	22.58	No	ns	0.9316	B-F	
shank3n vs. shank3n CBZ	10.43	No	ns	>0.9999	C-D	
shank3n vs. shank3c	-10.2	No	ns	>0.9999	C-E	
shank3n vs. shank3c CBZ	-13.82	No	ns	>0.9999	C-F	
shank3n CBZ vs. shank3c	-20.64	No	ns	>0.9999	D-E	
shank3n CBZ vs. shank3c CBZ	-24.25	No	ns	>0.9999	D-F	
shank3c vs. shank3c CBZ	-3.611	No	ns	>0.9999	E-F	
Test details	Mean rank 1	Mean rank 2	Mean rank diff.	n1	n2	Z
WT vs. WT cbz	127.8	102.1	25.72	35	37	2.141
WT vs. shank3n	127.8	65.68	62.12	35	19	4.278
WT vs. shank3n CBZ	127.8	55.25	72.55	35	24	5.373
WT vs. shank3c	127.8	75.89	51.91	35	27	3.978
WT vs. shank3c CBZ	127.8	79.5	48.3	35	34	3.937
WT cbz vs. shank3n	102.1	65.68	36.4	37	19	2.531
WT cbz vs. shank3n CBZ	102.1	55.25	46.83	37	24	3.507
WT cbz vs. shank3c	102.1	75.89	26.19	37	27	2.031
WT cbz vs. shank3c CBZ	102.1	79.5	22.58	37	34	1.866
shank3n vs. shank3n CBZ	65.68	55.25	10.43	19	24	0.6669
shank3n vs. shank3c	65.68	75.89	-10.2	19	27	0.6688
shank3n vs. shank3c CBZ	65.68	79.5	-13.82	19	34	0.9467
shank3n CBZ vs. shank3c	55.25	75.89	-20.64	24	27	1.444
shank3n CBZ vs. shank3c CBZ	55.25	79.5	-24.25	24	34	1.785
shank3c vs. shank3c CBZ	75.89	79.5	-3.611	27	34	0.2749

* p < 0.05,

** p < 0.01,

*** p < 0.001,

**** p < 0.0001.

**Table 34.** ANOVA of 30-second light-off for 5 μM MPEP-exposed WT, *shank3abN-/-*, and *shank3abC-/-* larvae. See Figure 6a.

Table analyzed	MPEP 30 sec lights-off
Kruskal-Wallis test	
P value	<0.001
Exact or approximate P value?	Approximate
P value summary	***
Do the medians vary signif. (P < 0.05)?	Yes
Number of groups	6
Kruskal-Wallis statistic	45.04
Data summary	
Number of treatments (columns)	6
Number of values (total)	153

* p < 0.05,

** p < 0.01,

*** p < 0.001,

**** p < 0.0001.

**Table 35.** Dunn’s multiple comparisons of 30-second lights-off for 5 μM MPEP-exposed WT, *shank3abN-/-*, and *shank3abC-/-* larvae. See Figure 6a.

Number of families	1					
Number of comparisons per family	15					
Alpha	0.05					
Dunn's multiple comparisons test	Mean rank diff.	Significant?	Summary	Adjusted P Value		
shank3+/+ DMSO vs. shank3+/+ MPEP	48.38	Yes	**	0.004	A-B	
shank3+/+ DMSO vs. shank3n DMSO	57.11	Yes	***	<0.001	A-C	
shank3+/+ DMSO vs. shank3n MPEP	65.55	Yes	***	<0.001	A-D	
shank3+/+ DMSO vs. shank3c DMSO	53.83	Yes	**	0.001	A-E	
shank3+/+ DMSO vs. shank3c MPEP	71.91	Yes	***	<0.001	A-F	
shank3+/+ MPEP vs. shank3n DMSO	8.724	No	ns	>0.99	B-C	
shank3+/+ MPEP vs. shank3n MPEP	17.17	No	ns	>0.99	B-D	
shank3+/+ MPEP vs. shank3c DMSO	5.45	No	ns	>0.99	B-E	
shank3+/+ MPEP vs. shank3c MPEP	23.53	No	ns	>0.99	B-F	
shank3n DMSO vs. shank3n MPEP	8.445	No	ns	>0.99	C-D	
shank3n DMSO vs. shank3c DMSO	-3.274	No	ns	>0.99	C-E	
shank3n DMSO vs. shank3c MPEP	14.8	No	ns	>0.99	C-F	
shank3n MPEP vs. shank3c DMSO	-11.72	No	ns	>0.99	D-E	
shank3n MPEP vs. shank3c MPEP	6.358	No	ns	>0.99	D-F	
shank3c DMSO vs. shank3c MPEP	18.08	No	ns	>0.99	E-F	
Test details	Mean rank 1	Mean rank 2	Mean rank diff.	n1	n2	Z
shank3+/+ DMSO vs. shank3+/+ MPEP	127.3	78.95	48.38	23	24	3.677
shank3+/+ DMSO vs. shank3n DMSO	127.3	70.23	57.11	23	33	4.864
shank3+/+ DMSO vs. shank3n MPEP	127.3	61.78	65.55	23	31	5.624
shank3+/+ DMSO vs. shank3c DMSO	127.3	73.5	53.83	23	19	3.967
shank3+/+ DMSO vs. shank3c MPEP	127.3	55.42	71.91	23	23	5.868
shank3+/+ MPEP vs. shank3n DMSO	78.95	70.23	8.724	24	33	0.682
shank3+/+ MPEP vs. shank3n MPEP	78.95	61.78	17.17	24	31	1.35
shank3+/+ MPEP vs. shank3c DMSO	78.95	73.5	5.45	24	19	0.3761
shank3+/+ MPEP vs. shank3c MPEP	78.95	55.42	23.53	24	23	1.774
shank3n DMSO vs. shank3n MPEP	70.23	61.78	8.445	33	30	0.7513
shank3n DMSO vs. shank3c DMSO	70.23	73.5	-3.274	33	19	0.2477
shank3n DMSO vs. shank3c MPEP	70.23	55.42	14.8	33	23	1.248
shank3n MPEP vs. shank3c DMSO	61.78	73.5	-11.72	31	19	0.8918
shank3n MPEP vs. shank3c MPEP	61.78	55.42	6.358	31	23	0.5399
shank3c DMSO vs. shank3c MPEP	73.5	55.42	18.08	19	23	1.322

* p < 0.05,

** p < 0.01,

*** p < 0.001,

**** p < 0.0001.

**Table 36.** ANOVA of 5-minute lights-off for 5 μM MPEP-exposed WT, *shank3abN-/-*, and *shank3abC-/-* larvae. See Figure 6b.

Table analyzed	shank3 MPEP 5 min lights-off
Kruskal-Wallis test	
P value	<0.0001
Exact or approximate P value?	Approximate
P value summary	****
Do the medians vary signif. (P < 0.05)?	Yes
Number of groups	6
Kruskal-Wallis statistic	27
Data summary	
Number of treatments (columns)	6
Number of values (total)	153

* p < 0.05,

** p < 0.01,

*** p < 0.001,

**** p < 0.0001.

**Table 37.** Dunn’s multiple comparisons of 5-minute lights-off for 5 μM MPEP-exposed WT, *shank3abN-/-*, and *shank3abC-/-* larvae. See Figure 6b.

Number of families	1					
Number of comparisons per family	15					
Alpha	0.05					
Dunn's multiple comparisons test	Mean rank diff.	Significant?	Summary	Adjusted P Value		
WT dmso vs. WT mpep	40.31	Yes	*	0.0225	A-B	
WT dmso vs. shk3n dmso	50.63	Yes	***	0.0003	A-C	
WT dmso vs. shk3 mpep	49.3	Yes	***	0.0006	A-D	
WT dmso vs. shk3c dmso	39.96	Yes	*	0.0473	A-E	
WT dmso vs. shk3c mpep	58.7	Yes	****	<0.0001	A-F	
WT mpep vs. shk3n dmso	10.32	No	ns	>0.9999	B-C	
WT mpep vs. shk3 mpep	8.994	No	ns	>0.9999	B-D	
WT mpep vs. shk3c dmso	-0.3502	No	ns	>0.9999	B-E	
WT mpep vs. shk3c mpep	18.4	No	ns	>0.9999	B-F	
shk3n dmso vs. shk3 mpep	-1.329	No	ns	>0.9999	C-D	
shk3n dmso vs. shk3c dmso	-10.67	No	ns	>0.9999	C-E	
shk3n dmso vs. shk3c mpep	8.074	No	ns	>0.9999	C-F	
shk3 mpep vs. shk3c dmso	-9.344	No	ns	>0.9999	D-E	
shk3 mpep vs. shk3c mpep	9.403	No	ns	>0.9999	D-F	
shk3c dmso vs. shk3c mpep	18.75	No	ns	>0.9999	E-F	
Test details	Mean rank 1	Mean rank 2	Mean rank diff.	n1	n2	Z
WT dmso vs. WT mpep	115.1	74.76	40.31	23	24	3.174
WT dmso vs. shk3n dmso	115.1	64.44	50.63	23	33	4.293
WT dmso vs. shk3 mpep	115.1	65.77	49.3	23	31	4.125
WT dmso vs. shk3c dmso	115.1	75.11	39.96	23	19	2.953
WT dmso vs. shk3c mpep	115.1	56.36	58.7	23	23	4.573
WT mpep vs. shk3n dmso	74.76	64.44	10.32	24	33	0.8869
WT mpep vs. shk3 mpep	74.76	65.77	8.994	24	31	0.7622
WT mpep vs. shk3c dmso	74.76	75.11	-0.3502	24	19	0.02614
WT mpep vs. shk3c mpep	74.76	56.36	18.4	24	23	1.449
shk3n dmso vs. shk3 mpep	64.44	65.77	-1.329	33	30	0.1228
shk3n dmso vs. shk3c dmso	64.44	75.11	-10.67	33	19	0.8508
shk3n dmso vs. shk3c mpep	64.44	56.36	8.074	33	23	0.6847
shk3 mpep vs. shk3c dmso	65.77	75.11	-9.344	31	19	0.7361
shk3 mpep vs. shk3c mpep	65.77	56.36	9.403	31	23	0.7868
shk3c dmso vs. shk3c mpep	75.11	56.36	18.75	19	23	1.385

* p < 0.05,

** p < 0.01,

*** p < 0.001,

**** p < 0.0001.

**Table 38.** Paired t-test for 3 mM PTZ-exposed *shank3abN-/-* larvae. See Figure 7b.

Table analyzed	3 mM PTZ
Column A	0.1% DMSO
vs.	vs.
Column B	PTZ
Test details	
Test name	Paired t test
Variance assumption	Individual variance for each group
Multiple comparisons	False Discovery Rate (FDR)
Method	Two-stage step-up (Benjamini, Krieger, and Yekutieli)
Desired FDR (Q)	0.20%
Number of tests performed	2
Number of rows omitted	0
Number of rows with incomplete data	1

**Table 39.** Paired t-test significance table for 3 mM PTZ-exposed *shank3abN-/-* larvae. See Figure 7b.

Column1	Discovery?	P value	Mean of 0.1% DMSO	Mean of PTZ	Diff.	SE of diff.	t ratio	df	q value
WT	Yes	<0.000001	829.5	3601	-2771	304.5	9.101	30	<0.000001
shk3n Hom	No	0.101513	1066	1538	-471.8	279.6	1.688	31	0.050858
	P value	Mean of 0.1% DMSO	Mean of PTZ	Difference	SE of difference	t ratio	df	q value	
WT	<0.000001	829.5	3601	-2771	304.5	9.101	30	<0.000001	

**Table 40.** Paired t-test for 3 mM PTZ-exposed *shank3abC-/-* larvae. See Figure 7b.

Table analyzed	3 mM PTZ
Column A	0.1% DMSO
vs.	vs.
Column B	PTZ
Test details	
Test name	Paired t test
Variance assumption	Individual variance for each group
Multiple comparisons	False Discovery Rate (FDR)
Method	Two-stage step-up (Benjamini, Krieger, and Yekutieli)
Desired FDR (Q)	0.20%
Number of tests performed	2
Number of rows omitted	0
Number of rows with incomplete data	2

**Table 41.** Paired t-test significance table for 3 mM PTZ-exposed *shank3abC-/-* larvae. See Figure 7b.

Column1	Discovery?	P value	Mean of 0.1%DMSO	Mean of PTZ	Difference	SE of difference	t ratio	df	q value
WT	Yes	<0.000001	951.5	3491	-2539	222.1	11.43	30	<0.000001
shk3c Hom	No	0.0581	770.5	1021	-450.4	150.4	2.995	27	0.002911
	P value	Mean of 0.1% DMSO	Mean of PTZ	Difference	SE of difference	t ratio	df	q value	
WT	<0.000001	951.5	3491	-2539	222.1	11.43	30	<0.000001	

## Data Availability

DRYAD: Drugs prescribed for Phelan-McDermid syndrome differentially impact sensory behaviors in shank3 zebrafish models. https://doi.org/10.5061/dryad.hqbzkh1kn ( Kozol & Dallman, 2023). DRYAD: ARRIVE checklist for ‘Drugs prescribed for Phelan-McDermid syndrome differentially impact sensory behaviors in shank3 zebrafish models.’ https://doi.org/10.5061/dryad.hqbzkh1kn ( Kozol & Dallman, 2023). Data are available under the terms of the Creative Commons Zero “No rights reserved” data waiver (CC0 1.0 Public domain dedication).
