# Supplementary material for: Drugs prescribed for Phelan-McDermid syndrome differentially impact sensory behaviors in shank3 zebrafish models
Source: F1000Res. 2023 Sep 27;12:84. Originally published 2023 Jan 23. [Version 2] doi: 10.12688/f1000research.127830.2 (PMC10589628; doi:10.12688/f1000research.127830.2)
Supplement: Peer review history

# Reviewer report: Reviewer response for version 2

**DOI:** 10.5256/f1000research.155643.r210229

**Published:** 2023-10-20

**Authors:** Sara Moir Sarasua

**Affiliations:** 1 Clemson University, Clemson, South Carolina, USA

I read the revisions and would like to change my review to Approve. I greatly appreciate the thoughtful responses for this very interesting and timely research.

Is the work clearly and accurately presented and does it cite the current literature?

Yes

If applicable, is the statistical analysis and its interpretation appropriate?

Yes

Are all the source data underlying the results available to ensure full reproducibility?

Yes

Is the study design appropriate and is the work technically sound?

Yes

Are the conclusions drawn adequately supported by the results?

Yes

Are sufficient details of methods and analysis provided to allow replication by others?

Yes

Reviewer Expertise:

NA

I confirm that I have read this submission and believe that I have an appropriate level of expertise to confirm that it is of an acceptable scientific standard.

# Reviewer report: Reviewer response for version 2

**DOI:** 10.5256/f1000research.155643.r210230

**Published:** 2023-09-29

**Authors:** Fumihito Ono

**Affiliations:** 1 Department of Physiology, Osaka Medical and Pharmaceutical University, Takatsuki, Japan

Authors addressed all of my concerns satisfactorily.

Is the work clearly and accurately presented and does it cite the current literature?

Yes

If applicable, is the statistical analysis and its interpretation appropriate?

Yes

Are all the source data underlying the results available to ensure full reproducibility?

Yes

Is the study design appropriate and is the work technically sound?

Yes

Are the conclusions drawn adequately supported by the results?

Yes

Are sufficient details of methods and analysis provided to allow replication by others?

Yes

Reviewer Expertise:

NA

I confirm that I have read this submission and believe that I have an appropriate level of expertise to confirm that it is of an acceptable scientific standard.

# Reviewer report: Reviewer response for version 1

**DOI:** 10.5256/f1000research.140374.r178147

**Published:** 2023-07-17

**Authors:** Sara Moir Sarasua

**Affiliations:** 1 Clemson University, Clemson, South Carolina, USA

Thank you for the opportunity to review this interesting and timely research. The authors present a study in which they use the zebrafish model of two shank3ab mutants (affecting either the N or C terminus of shank3a and shank3b) to measure sensory-motor behaviors by subjecting larvae to one of 3 medications for 24 hours and then measuring response to light/dark cycles.  The authors find that risperidone, but not LiCl or Carbamazepine, affects the response to light. They also find that the shank3ab models are resistant to the seizure-inducing drug PTZ. The methods presented will be helpful to others using the zebrafish model for Phelan-McDermid syndrome related research. This research is important because it models commonly used therapies in the zebrafish model with the hope of better understanding effective treatments in people with Phelan-McDermid syndrome due to *SHANK3* haploinsufficiency or *SHANK3* pathogenic variants. As such it will be of interest both to researchers investigating animal models as well as clinical researchers. With an interdisciplinary audience in mind, there are places where additional information would be most helpful.

 Our specific comments follow:

**Abstract:**

 The authors state in the Results that “risperidone normalized the VMR in shank3 models”. In the Results section, the authors state that “risperidone exacerbated hyporeactivity, but normalized hypoactivity.” Would the authors clarify?

**Introduction:**

 The authors provide a sound justification for the use of the zebrafish model in measuring sensory motor behavior.

 The authors do not indicate why PTZ trials were included in the experiments. This should be added to the introduction. Because epilepsy and seizures are important phenotypes in PMS, it is reasonable to look at this phenotype, thus it should be included in the introduction.

**Methods:**

 Page 3 (of the pdf version of the article). It would help the reader to state that these mutants include both shank3a and shank3b deletions of either the C or N terminus regions. Please direct the reader to the shank3a and shank3b model in Figure 1a. While this information is in the Figure, it is not mentioned in text. And because the figures and tables are located some distance from the text, the information is not as accessible as it could be.

 Table 1. It is somewhat unclear whether the “Effect on VMR” in this table derives from the cited literature or from the results of the research. After reading the manuscript, it would be helpful to have a table to summarize the results of the research in a table similar to Table 1, but based just on the present research results. A lot of work and many many statistical analyses were performed in this research study, and the results get somewhat lost. A summary table with an interpretation of what they mean would be very helpful to the reader.

 Page 4.

- What is the dosage used for the MS222 to euthanize the larvae?
- High-throughput behavioral screens – what is the outcome measured in the VMR experiments? Is it the same as described for the PTZ trials – total distance moved for 10 minutes?
- Please define in the methods how activity/hypoactivity was defined or measured. Please define how reactivity/hyporeactivity was defined. Does VMR include both of these measures? Or only the reactivity?

In the “Drug Screening” section, the system water needs more detail of quality measures i.e.. breakdown of the monitored levels of alkalinity, hardness, carbon dioxide, dissolved oxygen, pH, and anything else that is measured per-period or weekly.

 In the statistical analysis section, please tell the reader how the analyses were to be interpreted. By this we mean that normalization or rescue of a response was determined by comparing WT to mutant and finding no statistically significant difference, or a reduced difference. A challenge we discovered when interpreting the figures which display the statistical significance levels between WT-DMSO and mutants or WT drug and mutants, is that it was the lack of significance (lack of stars) that led to the interpretation of results. For instance, in Figure 3 describing response to risperidone, the caption says that risperidone normalizes the response. The reader can observe that the significant differences observed in the 30 second test, were no longer apparent in the 5 minute test. The 5 minute tests were similar between WT and mutants. We are so trained to look for the stars on graphs that it is hard to observe the lack of stars. Do the authors have suggestions to guide the reader in how to interpret these results? When the “biologically significant” finding is the non-statistically significant finding. It may also be of help to the reader to include a particular focus on the tables with statistical tests that much interest is given to such as the comparison of WT to mutant (showing difference) then WT to mutant-risp (showing no difference). Thus risperidone normalizing the outcome. Our first inclination was to suggest that the many tables be placed in a supplemental file, however, they are quite important. Is there a way to highlight these key comparisons that were used to come to the conclusions that the authors make?

 Table 2. Please tell the reader who is in Group A and who in Group B.

 Table 5, 2 ^nd^ to last row. Why does it say ABTL instead of WT? Elsewhere sometimes ABTL is used and sometimes WT. Are they interchangeable? I suggest using just WT if they are the same. The methods section tells the reader that WT is ABTL.

 Figures. We appreciate the authors defining what is depicted in the box plots and inclusion of the sample sizes.

 Figure 2. Please label the 2 columns of the graph. Two graphs are shown for each drug. One for distance (mm/30 sec) and the other Distance (mm/5min). It would help the reader to label the two columns/graphs. It is defined in Figure 3 and would help the reader to define it in Figure 2. It would help the reader to label each column (Activity) and (Reactivity) so the reader more clearly understands which graph shows which outcome.

 Figures 3, 4, 5. The figures show statistical comparisons between WT DMSO vs. the 4 shank3ab genotypes and WT drug vs. the 4 shank3ab genotypes by indicating with stars whether the comparisons are statistically significant. However, it is the absence of a statistical difference that leads to the interpretation. This is a challenge in reading the graphs and requires a detailed review of the statistical tables. Do the authors have any suggestions?

 Figure 4. In this figure caption, it reads “reactivity or activity” rather than “hyporeactivity or hypoactivity” as in the previous figure. It helps the reader to be consistent in use of the terms across the written portion as well as figures/tables.

 Figure 7.  panel b and c, label WT and N-/+ and N -/-. In panel a, it appears the investigators assessed N-/- and N-/+. However, in the accompanying graphics, it appears only the N-/- is graphed. Is that correct? Is N-/+ a heterozygote? I do not see assessment of heterozygotes in the methods or anywhere else in the manuscript. Please include the methods in the methods section.

 Page 26. It is understandable to model the strongest phenotypic response in the homozygous models. However, PMS is caused by a heterozygous deletion/variant in *SHANK3*. Would the authors comment on how their results might be applied to the case of heterozygous mutations and how this work translates to the human clinical condition? Would the authors also comment on how the fish paralogs shank3a and shank3b relate to the human *SHANK3*?

 It would help the reader if the authors would help interpret the direction of effect that is to the benefit. For instance, if treated with risperidone, the activity in the mutants is less than when not treated with risperidone. The authors say this exacerbated the hyporeactivity. For clinical usage, is increased hyporeactivity (is that less reactivity?) a desired outcome? If treatment with risperidone normalized activity, is that a desired outcome? Back to an earlier comment, it would help the reader to provide a summary table telling the reader, for each drug and each genotype, what the effect was (no difference, Decreased activity, Increased activity, Normalized compared to WT, decreased reactivity, Increased reactivity, Normalized reactivity, And which direction is the desired direction)

 What does this study add to what was previously known?

**General points**

 The flow of the manuscript is disrupted with the many figures and tables and the text is sometimes lost among the tables. It would help to have the Figures and Tables placed where referenced and include them all within the Results section rather than interspersed.

**Discussion**

 How do the authors interpret the genotype-specific findings?

Is the work clearly and accurately presented and does it cite the current literature?

Yes

If applicable, is the statistical analysis and its interpretation appropriate?

Yes

Are all the source data underlying the results available to ensure full reproducibility?

Yes

Is the study design appropriate and is the work technically sound?

Yes

Are the conclusions drawn adequately supported by the results?

Yes

Are sufficient details of methods and analysis provided to allow replication by others?

Yes

Reviewer Expertise:

Phelan-McDermid syndrome

I confirm that I have read this submission and believe that I have an appropriate level of expertise to confirm that it is of an acceptable scientific standard, however I have significant reservations, as outlined above.

# Response

**Published:** 2023-09-06

**Authors:** Julia Dallman

**Affiliations:** University of Miami, USA

*First, we would like to thank Dr. Sarasua for her thorough and clear feedback for how to improve the presentation of our work. We respond (our responses are in italics) to each suggestion below.*

 Our specific comments follow:

**Abstract:**

 The authors state in the Results that “risperidone normalized the VMR in shank3 models”. In the Results section, the authors state that “risperidone exacerbated hyporeactivity, but normalized hypoactivity.” Would the authors clarify?

*Response: We agree that as written there seems to be a discrepancy between what is stated in the abstract and results/discussion. We decided not to include the terms and definitions of hypoactivivity and hyporeactivity in the abstract due to the need for brevity but in the current version, we edited the text in the abstract results section to read* *“* *We found that risperidone* ***partially*** *normalized the VMR in shank3 models.”*

**Introduction:**

 The authors provide a sound justification for the use of the zebrafish model in measuring sensory motor behavior.

 The authors do not indicate why PTZ trials were included in the experiments. This should be added to the introduction. Because epilepsy and seizures are important phenotypes in PMS, it is reasonable to look at this phenotype, thus it should be included in the introduction.

*Response: We edited the following sentence to more clearly introduce PTZ experiments “* *Lastly, we quantified swimming before and after exposure to  pentylenetetrazole (PTZ), a drug used in animal models to* *better understand susceptibility to seizures, at doses that normally cause hyperactivity in wild type larvae* *(Baraban et al., 2005; Hoffman et al., 2016; Liu and Baraban, 2019)* *.*

**Methods:**

 Page 3 (of the pdf version of the article). It would help the reader to state that these mutants include both shank3a and shank3b deletions of either the C or N terminus regions. Please direct the reader to the shank3a and shank3b model in Figure 1a. While this information is in the Figure, it is not mentioned in text. And because the figures and tables are located some distance from the text, the information is not as accessible as it could be.

*Response:* *We agree that more information on the design of our mutations provides clarity for the reader. We have since added the text, “Readers should note that each model includes a mutation in both the a and the b ohnolog of the shank3 gene and therefor mutants are referred to as shank3ab; mutations in shank3abN are located near the N-terminus while those in shank3abC are located near the C-terminus of the predicted Shank3 protein product (Figure 1a).”*

 Table 1. It is somewhat unclear whether the “Effect on VMR” in this table derives from the cited literature or from the results of the research. After reading the manuscript, it would be helpful to have a table to summarize the results of the research in a table similar to Table 1, but based just on the present research results. A lot of work and many many statistical analyses were performed in this research study, and the results get somewhat lost. A summary table with an interpretation of what they mean would be very helpful to the reader.

*Response: We agree with the reviewer that clarity on the content of table 1 would help direct the reader’s attention to the important summaries from our study. This table includes the drug, drug classification, receptors that the drug targets, the summary of how the drug effected VMR in our study and references that provided mechanism of action and/or helped us determine dosages to test for our dose response assays. We have since edited the last sentence of the introduction to read* *“* *Results of the above experiments are summarized in the column entitled ‘effect on VMR’ in Table 1.” And* *the table description to read,* *“Drugs used in this study are listed to the left followed by indication and target(s)/mechanism of action. These are based on relevant references in the rightmost column. Drug effects on VMR in are based results from this study.”*

 Page 4.

- What is the dosage used for the MS222 to euthanize the larvae?

*Response: We thank the reviewer for finding this concentration omission. We have since added to the text, “200 mg/L”.*

  

- High-throughput behavioral screens – what is the outcome measured in the VMR experiments? Is it the same as described for the PTZ trials – total distance moved for 10 minutes?

*Response: We thank the reviewer for finding this omission in the methods. We have added the definition of activity and reactivity, along with the measurement units that were compared. The text now reads, “* *Behavior was analyzed by binning the raw ethovision movement data into 30 second and 5 minute bins. We then defined behaviors in the first 30 seconds after dark transitions as reactivity and behaviors sustained  across the full five minutes of darkness as activity.* *”*

  

- Please define in the methods how activity/hypoactivity was defined or measured. Please define how reactivity/hyporeactivity was defined. Does VMR include both of these measures? Or only the reactivity?

*Response: We thank the reviewer for finding this omission in the methods. We have added the definition of hyper- and hypo- following the above mentioned definition of baseline activity and reactivity. The manuscript now reads, “* *Therefore, a statistical increase or decrease in swimming during the first 30 seconds was defined as hyperreactive or hyporeactive respectively; a statistical increase or decrease in swimming during the full five minutes was defined as hyperactive or hypoactive respectively.* *”*

 In the “Drug Screening” section, the system water needs more detail of quality measures i.e.. breakdown of the monitored levels of alkalinity, hardness, carbon dioxide, dissolved oxygen, pH, and anything else that is measured per-period or weekly.

*Thank you for pointing out this oversight. We have added the following to the methods section.* “ *The water in which the adult fish are housed are tested for pH and conductivity by probes that are always sampling, ‘system water’. System water is tap water that goes through a water softener, a charcoal filter, and reverse osmosis membranes to make the water less hard/alkaline, remove contaminants and ions respectively. This purified water is stored on a 100 gallon storage tank and used for 10% daily water exchanges that are controlled by a solenoid. pH 7.0-8.1 and conductivity 350-800 µS are kept within range by two dosers, one with sodium bicarbonate (pH) and the other with instant ocean (conductivity). We also track room humidity and temperature on a daily basis. These values are important to track because the temperature of the water is regulated by air temperature.*”

 In the statistical analysis section, please tell the reader how the analyses were to be interpreted. By this we mean that normalization or rescue of a response was determined by comparing WT to mutant and finding no statistically significant difference, or a reduced difference. A challenge we discovered when interpreting the figures which display the statistical significance levels between WT-DMSO and mutants or WT drug and mutants, is that it was the lack of significance (lack of stars) that led to the interpretation of results. For instance, in Figure 3 describing response to risperidone, the caption says that risperidone normalizes the response. The reader can observe that the significant differences observed in the 30 second test, were no longer apparent in the 5 minute test. The 5 minute tests were similar between WT and mutants. We are so trained to look for the stars on graphs that it is hard to observe the lack of stars. Do the authors have suggestions to guide the reader in how to interpret these results? When the “biologically significant” finding is the non-statistically significant finding. It may also be of help to the reader to include a particular focus on the tables with statistical tests that much interest is given to such as the comparison of WT to mutant (showing difference) then WT to mutant-risp (showing no difference). Thus risperidone normalizing the outcome. Our first inclination was to suggest that the many tables be placed in a supplemental file, however, they are quite important. Is there a way to highlight these key comparisons that were used to come to the conclusions that the authors make?

*Response: We agree that it might be confusing to have the lack of significance indicate rescue. We have edited the figure legend to now state, “* *No statistical significance between drug exposed mutant and control was interpreted as normalization.*”

 Table 2. Please tell the reader who is in Group A and who in Group B.

*Response: We agree with the reviewer that these labels were arbitrary and did not define the categories analyzed. We have updated these labels to “lights-on” for “Group A” and “lights-off” for “Group B”.*

 Table 5, 2 ^nd^ to last row. Why does it say ABTL instead of WT? Elsewhere sometimes ABTL is used and sometimes WT. Are they interchangeable? I suggest using just WT if they are the same. The methods section tells the reader that WT is ABTL.

*Response: We agree with the reviewer that multiple names is confusing. We have since taken ABTL out of all instances, besides the initial definition of WT in the “Ethics, fish maintenance and husbandry” section.*

 Figures. We appreciate the authors defining what is depicted in the box plots and inclusion of the sample sizes.

*Response: We thank the reviewer for the compliment.*

 Figure 2. Please label the 2 columns of the graph. Two graphs are shown for each drug. One for distance (mm/30 sec) and the other Distance (mm/5min). It would help the reader to label the two columns/graphs. It is defined in Figure 3 and would help the reader to define it in Figure 2. It would help the reader to label each column (Activity) and (Reactivity) so the reader more clearly understands which graph shows which outcome.

*Response: Thank you for this suggesting this change. We have revised both Figure 1 and Figure 2 accordingly.*

 Figures 3, 4, 5. The figures show statistical comparisons between WT DMSO vs. the 4 shank3ab genotypes and WT drug vs. the 4 shank3ab genotypes by indicating with stars whether the comparisons are statistically significant. However, it is the absence of a statistical difference that leads to the interpretation. This is a challenge in reading the graphs and requires a detailed review of the statistical tables. Do the authors have any suggestions?

*Response: We agree with the reviewer that the submitted version did not explicitly state how to interpret the graphs. We have since edited the figure legends of Figures 4-5 to read, “No statistical significance between drug exposed mutant and control was interpreted as normalization.”*

 Figure 4. In this figure caption, it reads “reactivity or activity” rather than “hyporeactivity or hypoactivity” as in the previous figure. It helps the reader to be consistent in use of the terms across the written portion as well as figures/tables.

*Response: Our figure legend title, “Figure 4. LiCl does not impact lights-off activity or reactivity behaviors in wildtype and shank3ab mutants.” Referred to the finding that no behavior changed when exposed to LiCL, wildtype or mutant. We have tried to address this issue by changing the figure legend title to be, “Figure 4. LiCl does not impact lights-off VMR behaviors in wildtype and shank3ab mutants.”*

 Figure 7.  panel b and c, label WT and N-/+ and N -/-. In panel a, it appears the investigators assessed N-/- and N-/+. However, in the accompanying graphics, it appears only the N-/- is graphed. Is that correct? Is N-/+ a heterozygote? I do not see assessment of heterozygotes in the methods or anywhere else in the manuscript. Please include the methods in the methods section.

*Response: We agree that the figure is confusing when seeing the inconsistencies in genotype titles for the image and graph. We decided to focus on the homozygote data, because heterozygotes were not tested or analyzed for the other drugs used in the study. We have edited the text for the “Drug Screening” section to now include clarity about our analysis. The text now reads, “* *Both heterozygote and homozygote larvae were tested for seizure susceptibility, however to remain consistent with the other genotypes analyzed in the study, we chose to focus on the homozygotic data for statistical analyses to be consistent with the rest of the paper* *.”*

 Page 26. It is understandable to model the strongest phenotypic response in the homozygous models. However, PMS is caused by a heterozygous deletion/variant in *SHANK3*. Would the authors comment on how their results might be applied to the case of heterozygous mutations and how this work translates to the human clinical condition? Would the authors also comment on how the fish paralogs shank3a and shank3b relate to the human *SHANK3*?

*Response: We assessed heterozygotes in previous research for VMR and other phenotypes, see Kozol et al. 2015 Human Molecular Genetics, James et al. 2019 Molecular Autism, and Kozol et al. 2021 Nature Communications Biology. In our previous work, we found that the homozygotes provided the most pronounced version of a phenotype and therefore an ideal choice for determining how drugs impact these phenotypes. Because homozygotes are viable in zebrafish, we believe the model provides an opportunity to screen a relatively strong phenotype that is qualitatively similar to the heterozygous phenotype for drug rescue that could be translatable for genetic forms of ASD. For instance, recently a clinical assessment of individuals with PMS found that a large minority suffer visual hyporeactivity, see Walinga et al. 2022, European Journal of Medical Genetics. Therefore, our shank3 gene models may provide a phenotype that is similar to visually initiated hyporeactive symptoms present in individuals with shank3 variant PMS. We believe further research determining secondary effects of commonly prescribed drugs for any condition would benefit the knowledgebase of clinical researchers and doctors.*

*In terms of the relationship between human and zebrafish gene paralogs, we previously published a study that has an analysis that describes how conserved shank3 genes are in fish. We found that both ohnologs are highly conserved with humans, with both copies maintaining all of the major conserved protein interacting domains. Finally, we found shank3a was more highly conserved, with higher percent pairwise comparisons and inclusion of smaller single protein interacting domains. See Kozol et al. 2015, Human Molecular Genetics for more details.*

 It would help the reader if the authors would help interpret the direction of effect that is to the benefit. For instance, if treated with risperidone, the activity in the mutants is less than when not treated with risperidone. The authors say this exacerbated the hyporeactivity. For clinical usage, is increased hyporeactivity (is that less reactivity?) a desired outcome? If treatment with risperidone normalized activity, is that a desired outcome? Back to an earlier comment, it would help the reader to provide a summary table telling the reader, for each drug and each genotype, what the effect was (no difference, Decreased activity, Increased activity, Normalized compared to WT, decreased reactivity, Increased reactivity, Normalized reactivity, And which direction is the desired direction)

*Response: We understand that our original interpretation of the relationship between our results and how they relate to the human condition was not clear. This study was looking to see how drugs prescribed for one symptom, such as aggressive behavior or epilepsy, impacted sensory perception, with a focus on sensory hyporeactivity that is found in our models. Therefore, our results provide evidence that many non-selective molecules used to treat core symptoms, can in reality exacerbate or help medicate sensory symptoms that may not have been tested yet in a clinical study. We strongly believe that normalizing or medicating a sensory deficit to baseline should be a primary goal for a clinician, seeing that sensory deficits are one of several quality of life ailments for individuals with PMS. Finally, table 1 provides the primary results for each drugs effect on VMR and we have provided clarity in the text to direct the reader to this information. See previous response.*

 What does this study add to what was previously known?

*Response: As stated above, this study provides evidence that many commonly prescribed drugs for core symptoms of Phelan-Mcdermid Syndrome may be unintentionally impacting sensory symptoms, whether positively (risperidone) or negatively.*

**General points**

 The flow of the manuscript is disrupted with the many figures and tables and the text is sometimes lost among the tables. It would help to have the Figures and Tables placed where referenced and include them all within the Results section rather than interspersed.

*Response: We understand the frustration for the reader that the layout of the paper makes the manuscript hard to read. However, we disagree and would prefer that the tables be added after the main body and before the references. This makes the reading flow easiest visually and maintains the tables for readers to reference if they are interested in specific metrics not mentioned in the text or figure legends, such as t and p values.*

# Reviewer report: Reviewer response for version 1

**DOI:** 10.5256/f1000research.140374.r164132

**Published:** 2023-03-09

**Authors:** Fumihito Ono

**Affiliations:** 1 Department of Physiology, Osaka Medical and Pharmaceutical University, Takatsuki, Japan

This paper by Kozol and Dallman studied the effects of small molecule drugs on the behavior of shank3 mutant zebrafish, using it as a model of PMS. Specifically, authors examined their response to the change of illumination. The experiments were reasonably designed and the analysis was rigorously executed. Small molecules examined in the study also have clinical significance.

 I have several suggestions listed below.

- Please provide the definition of “activity” and “reactivity” in Introduction or Methods. I presumed that the former corresponds to the overall activity during the dark 5 min while the latter to that of first 30 seconds. Because this concept is central to the study design and the analysis, it may be worthwhile to highlight it in Fig.1.
- The difference of reactivity and activity between N-term and C-term mutants in CBZ is intriguing. Is it known whether human patients with either mutation show distinct response to CBZ?
- Concentration of each drug in the bath solution was determined by making a dose response curve first in Wild type fish (Fig.2). The concentration in the CNS may reach that of the bath solution depending on the chemical property of the drug, while some drugs may display disparity between CNS and bath. It will be difficult to directly measure the concentration of each drug in the brain. Therefore, I suggest adding some discussion on pharmacokinetics (absorption, excretion, blood brain barrier etc.) in the text.
- In Fig.1c and d, please put labels on the horizontal axes. While readers can identify three groups based on their colors, labeling will make the identification easier.
- In Fig.2C, please include data for 200 microM.
- If allowed in the journal format, please consider moving most of the tables to supplement. It is definitely good that readers can directly look at all the analysis. On the other hand, having 41 tables in the middle of the main text is somewhat distracting.

Is the work clearly and accurately presented and does it cite the current literature?

Yes

If applicable, is the statistical analysis and its interpretation appropriate?

Yes

Are all the source data underlying the results available to ensure full reproducibility?

Yes

Is the study design appropriate and is the work technically sound?

Yes

Are the conclusions drawn adequately supported by the results?

Yes

Are sufficient details of methods and analysis provided to allow replication by others?

Yes

Reviewer Expertise:

NA

I confirm that I have read this submission and believe that I have an appropriate level of expertise to confirm that it is of an acceptable scientific standard, however I have significant reservations, as outlined above.

# Response

**Published:** 2023-09-05

**Authors:** Julia Dallman

**Affiliations:** University of Miami, USA

*First, we would like to thank Dr. Ono for his careful and insightful review of our paper. We respond (our responses are in italics) to each suggestion below.*

- Please provide the definition of “activity” and “reactivity” in Introduction or Methods. I presumed that the former corresponds to the overall activity during the dark 5 min while the latter to that of first 30 seconds. Because this concept is central to the study design and the analysis, it may be worthwhile to highlight it in Fig.1.

*Response: We agree with the reviewer that we omitted the definitions for activity and reactivity. We have since edited the text of both the introduction and the methods to read,*

*Introduction:* *”* *During the VMR, sudden changes in illumination from light to dark evoke abrupt increases in swimming behavior as larvae search the well for a way to return to the light (Horstick et al. 2017); we capture the abrupt response by quantifying swimming in the first 30 seconds right after the transition to dark, referred to hereafter as reactivity, but the larvae sustain their search for the full 5 minutes, referred to hereafter as activity”.*

*Methods: “Behavior was analyzed by binning the raw ethovision movement data into 30 second and 5 minute bins. We then defined behaviors in the first 30 seconds after dark transitions as reactivity and behaviors sustained  across the full five minutes of darkness as activity. Therefore, a statistical increase or decrease in swimming during the first 30 seconds was defined as hyperreactive or hyporeactive respectively; a statistical increase or decrease in swimming during the full five minutes was defined as hyperactive or hypoactive respectively”*

  

- The difference of reactivity and activity between N-term and C-term mutants in CBZ is intriguing. Is it known whether human patients with either mutation show distinct response to CBZ?

*Response: We appreciate the reviewer’s curiosity in how our results relate to the literature. We know that CBZ is used to treat intractable epilepsy in some individuals with PMS. However, information regarding medication is usually a portion of meta analyses for clinical reviews or perspectives. Therefore, they do not indicate how CBZ impacts other symptoms of the patients.*

  

- Concentration of each drug in the bath solution was determined by making a dose response curve first in Wild type fish (Fig.2). The concentration in the CNS may reach that of the bath solution depending on the chemical property of the drug, while some drugs may display disparity between CNS and bath. It will be difficult to directly measure the concentration of each drug in the brain. Therefore, I suggest adding some discussion on pharmacokinetics (absorption, excretion, blood brain barrier etc.) in the text.

*Response:* *Thank you for pointing this out. We have added a paragraph to the discussion:*

*“* *It should be noted that the concentration of the drug reported here corresponds to that in the bath solution surrounding the larvae. This bath concentration may differ substantially from the brain concentration due to differences in on a drug by drug basis related to pharmacokinetic differences. These include the drug’s ability to cross the skin and/or blood brain barrier as well as differences in kinetics of drug breakdown and excretion* *(Skiba et al., 2023; Windell et al., 2023)* *. Also, this route of administration differs fundamentally from that in people and therefore drug dosage in zebrafish does not correspond to that in people.”*


* *

- In Fig.1c and d, please put labels on the horizontal axes. While readers can identify three groups based on their colors, labeling will make the identification easier.

*Response: We have added labels to the bottom of the box plots in Figure 1.*

  

- In Fig.2C, please include data for 200 microM.

*Response: We apologize for the error in labeling our graph. The initial submission has an incorrect dose response that has now been changed to, 80, 120 and 200 microM. The numbers in the methods, figure legend and statistical tables are all correct and have remained the same.*

  

- If allowed in the journal format, please consider moving most of the tables to supplement. It is definitely good that readers can directly look at all the analysis. On the other hand, having 41 tables in the middle of the main text is somewhat distracting.

*Response: We agree with the reviewer and have moved the tables detailing statistics to the end of the paper that comes just before the references.*
